# The material properties of a bacterial-derived biomolecular condensate tune biological function in natural and synthetic systems

**DOI:** 10.1038/s41467-022-33221-z

**Published:** 2022-09-26

**Authors:** Keren Lasker, Steven Boeynaems, Vinson Lam, Daniel Scholl, Emma Stainton, Adam Briner, Maarten Jacquemyn, Dirk Daelemans, Ashok Deniz, Elizabeth Villa, Alex S. Holehouse, Aaron D. Gitler, Lucy Shapiro

**Affiliations:** 1 Department of Developmental Biology, Stanford University School of Medicine, Stanford, CA USA; 2 Department of Integrative Structural and Computational Biology, The Scripps Research Institute, La Jolla, CA USA; 3 Department of Genetics, Stanford University School of Medicine, Stanford, CA USA; 4 Department of Molecular Biology, School of Biological Sciences, University of California San Diego, La Jolla, CA USA; 5 Clem Jones Centre for Ageing Dementia Research (CJCADR), Queensland Brain Institute (QBI), The University of Queensland, Brisbane, QLD Australia; 6 KU Leuven Department of Microbiology, Immunology, and Transplantation, Laboratory of Virology and Chemotherapy, Rega Institute, KU Leuven, Leuven, Belgium; 7 Howard Hughes Medical Institute, University of California San Diego, La Jolla, CA USA; 8 Department of Biochemistry and Molecular Biophysics, Washington University in St. Louis, St. Louis, MO USA; 9 Center for Science and Engineering of Living Systems (CSELS), Washington University in St. Louis, St. Louis, MO USA

**Keywords:** Intrinsically disordered proteins, Cellular microbiology, Synthetic biology

## Abstract

Intracellular phase separation is emerging as a universal principle for organizing biochemical reactions in time and space. It remains incompletely resolved how biological function is encoded in these assemblies and whether this depends on their material state. The conserved intrinsically disordered protein PopZ forms condensates at the poles of the bacterium *Caulobacter crescentus*, which in turn orchestrate cell-cycle regulating signaling cascades. Here we show that the material properties of these condensates are determined by a balance between attractive and repulsive forces mediated by a helical oligomerization domain and an expanded disordered region, respectively. A series of PopZ mutants disrupting this balance results in condensates that span the material properties spectrum, from liquid to solid. A narrow range of condensate material properties supports proper cell division, linking emergent properties to organismal fitness. We use these insights to repurpose PopZ as a modular platform for generating tunable synthetic condensates in human cells.

## Abstract (web summary)

“Intracellular phase separation is emerging as a universal principle for organizing biochemical reactions in time and space. Here the authors show that PopZ condensate dynamics support cell division and using PopZ modular architecture, the tunable PopTag platform was developed to enable designer condensates.”

## Introduction

Biomolecular condensation is a powerful mechanism underlying cellular organization and regulation in physiology and disease^1–3^. Many of these condensates are formed via reversible phase separation^2,4^, which allows for rapid sensing of and response to a range of cellular challenges^5,6^. Biomolecular condensates can adopt a broad spectrum of material properties, ranging from highly dynamic liquids to semi-fluid gels, glasses, and solid aggregates^4,7–10^. Perturbing protein condensation can alter organismal fitness^11–16^, and mutations promoting protein aggregation and other pathological phase transitions have been implicated in human disease^9,17–21^. Further, recent studies show that disrupting the fluidity of a biomolecular condensate can affect its function^22,23^. However, mechanistic studies on how condensate function is tuned along the entire width of the material properties spectrum remain lacking. Addressing this question is crucial to understanding how function is encoded into these condensates and how their material properties relate to biological fitness.

**Fig. 1:**
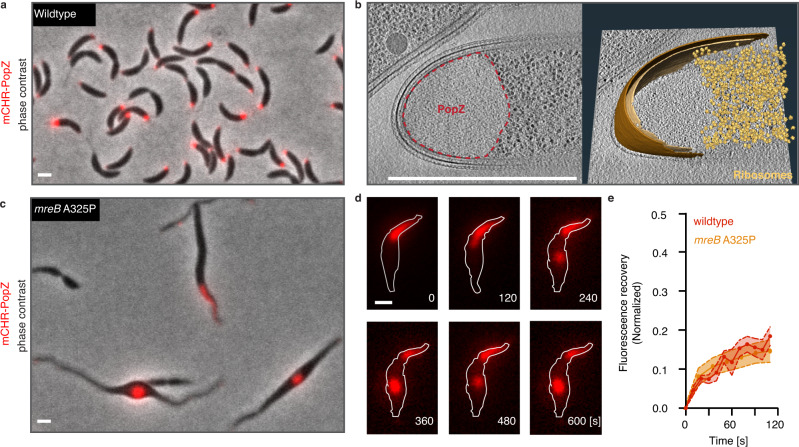
PopZ phase separates in *Caulobacter crescentus*. **a** PopZ self-assembles at the poles of wildtype *Caulobacter* cells. A fluorescent image of Δ*popZ Caulobacter* cells expressing mCherry-PopZ (red) from the *xylX* promoter on a high copy plasmid overlaid on a corresponding phase-contrast image. Scale bar, 1 μm. **b** The PopZ microdomain excludes ribosomes and forms a sharp convex boundary. (left) Slice through a tomogram of a cryo-ET focused ion beam-thinned Δ*popZ Caulobacter* cell overexpressing mCherry-PopZ. A dashed red line shows the boundaries of the PopZ region. (right) Segmentation of the tomogram in (left) showing the outer membrane (dark brown), inner membrane (light brown), and ribosomes (gold). Scale bar, 1 μm. **c**, **d** PopZ creates droplets in deformed *Caulobacter* cells. **c** A fluorescent image of *Caulobacter* cells bearing a *mreB* A325P mutant, expressing mCherry-PopZ (red) from the *xylX* promoter on a high copy plasmid overlaid on a corresponding phase-contrast image. Scale bar, 1 μm. **d** Fluorescent images show the PopZ microdomain (red) extending into the cell body, concurrent with the thinning of the polar region, producing a droplet that dynamically moves throughout the cell. Frames are 2 min apart. Scale bar, 1 μm**. e** PopZ dynamics are not affected by a release from the cell pole. Recovery following targeted photobleaching of a portion of an extended PopZ microdomain in wildtype and *mreB* A325P mutant cells. Cells expressing mCherry-PopZ from a high copy plasmid were imaged for 12 frames of laser scanning confocal microscopy following targeted photobleaching with high-intensity 561 nm laser light. Shown is the mean ± SEM of the normalized fraction of recovered signal in the bleached region; *n* equals 15 cells.

The bacterium *Caulobacter crescentus* reproduces by asymmetric division^24^, an event orchestrated by the intrinsically disordered Polar Organizing Protein Z (PopZ)^25,26^. PopZ self-assembles into 200 nm microdomains localized to the cell poles (Fig. 1a) and forms a homogeneous membraneless microdomain that excludes large protein complexes, such as ribosomes^27,28^ (Fig. 1b). PopZ is required for the formation of these polar microdomains as knock-out of the *popZ* gene results in their complete loss^27^. PopZ binds to at least 13 cell-cycle regulating proteins^29^ and selectively recruits them to the cell pole. Among them are members of the kinase-signaling cascades that control asymmetric cell division through spatial regulation of transcriptional programs^30^. Previous work has shown that PopZ mutants unable to self-assemble into a polar microdomain result in severe cell division defects^31^. This well-defined and important physiological function of the PopZ microdomain makes it an ideal system for studying how biological function is encoded into membraneless assemblies.

In this study, we show that PopZ phase separation underlies microdomain formation. By studying the molecular logic of this scaffold protein, we found that its emergent material properties are dictated by the sum of attractive and repulsive forces, mediated by an intrinsically disordered and a helical oligomerization domain, respectively. This framework allowed us to scan the spectrum of material properties with rationally designed PopZ mutants by tuning these attractive and repulsive forces. We identified a region of PopZ material properties that allow for optimal growth, therefore providing a unique case study on how selective pressures have tuned condensate features to maximize biological fitness. Lastly, we use our insights into the modular nature of the PopZ scaffold to create a condensation module for the generation of tunable condensates in eukaryote systems.

## Results

### PopZ undergoes phase separation in vitro and in vivo

**Fig. 2:**
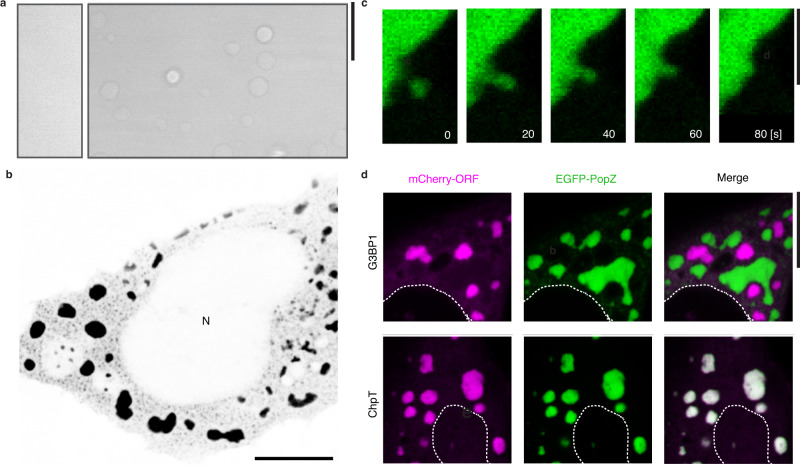
PopZ phase separates in vitro and in human U2OS cells. **a** The PopZ protein forms droplets in vitro in the presence of magnesium. Differential interference contrast microscopy images of PopZ at physiological concentration of 5 µM^30^ in 5 mM sodium phosphate at pH 6.0 with either 2 mM MgCl2 (left) or 5 mM MgCl2 (right). **b** *Caulobacter* PopZ expressed in human U2OS cells forms phase-separated condensates (black) in the cytoplasm but not the nucleus (N). **c** In vivo fusion and growth of PopZ condensates in human U2OS cells. 80 s time-lapse images of a small PopZ condensate (green) merging with a large PopZ condensate. Scale bar, 5 μm. **d** PopZ expressed in human U20S cells retains selectivity. (Top) EGFP-PopZ (green) and stress granule protein mCherry-G3BP1 (purple) form separate condensates. (Bottom) EGFP-PopZ (green) recruits the *Caulobacter* phosphotransfer protein mCherry-ChpT (magenta) when co-expressed in human U2OS cells. Scale bar, 10 μm.

To probe the dynamic behavior of PopZ, we expressed mCherry-tagged PopZ in a strain of *Caulobacter* bearing the *mreB*^A325P^ mutation^32^, which leads to irregular cellular elongation with thin polar regions and wide cell bodies^33^. While PopZ normally resides at the cell pole, in the *mreB*^A325P^ background, the microdomain deforms and extends into the cell body before undergoing fission, producing spherical droplets that move throughout the cell (Fig. 1c, d, Supplementary Fig. 1a). The deformation of the microdomain at the thinning cell pole and the minimization of surface tension when localized to the cytoplasm, and hence unrestrained by the plasma membrane, provide in vivo evidence that the PopZ microdomain has liquid-like properties. This observation is further supported by the partial fluorescence recovery of PopZ upon photobleaching, which indicates slow internal dynamic rearrangements^30^ (Fig. 1e). To test whether the PopZ protein is sufficient to drive condensate formation, we studied its behavior in vitro. Recombinant PopZ protein spontaneously demixed to form droplets at physiological concentrations (Supplementary Note) in the presence of divalent cations (Fig. 2a, Supplementary Fig. 1b–f).

PopZ is only found in α-proteobacteria. Additionally, the sequence composition of its intrinsically disordered region (IDR), a region often found in phase separating proteins^34,35^, is divergent from the human disordered proteome (Supplementary Fig. 2). We, therefore, reasoned that human cells could serve as an orthogonal system for studying PopZ condensation outside of the context of its *Caulobacter* binding clients. When expressed in a human osteosarcoma U2OS cell line, PopZ formed micron-sized cytoplasmic condensates (Fig. 2b) that underwent spontaneous fusion events (Fig. 2c) and displayed dynamic internal rearrangements, as assayed by FRAP. Importantly, even though expressed in human cells, PopZ condensates retained specificity for their bacterial client proteins, such as ChpT^30^, and were distinct from human stress granules (Fig. 2d). Thus, PopZ is sufficient for condensation and client recruitment, and human cells serve as an independent platform to study its behavior.

### PopZ IDR tunes the microdomain internal dynamics

**Fig. 3:**
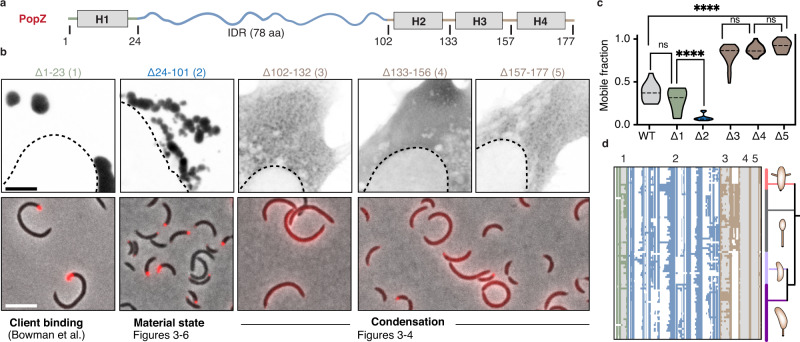
Domain organization of the PopZ condensate. **a** Domain organization of the PopZ protein from *Caulobacter crescentus*. PopZ is composed of a short N-term region with a predicted helix, H1 (gray box), a 78 amino-acid intrinsically disordered region (IDR, blue curly line), and a C-term region with three predicted helices, H2, H3, H4 (gray boxes). **b**. Region deletion and its effect on PopZ condensation. (top) EGFP fused to five PopZ deletions (black) expressed in human U2OS cells. (bottom) mCherry fused to four PopZ deletions (Δ1–23, Δ24–101, Δ102–132, and Δ133–177) (red) expressed in *ΔpopZ Caulobacter* cells. Scale bar, 10 μm **c**. Region deletion and its effect on PopZ mobility. FRAP, shown as mobile fractions, the plateau of the FRAP curves for the wildtype (gray), for the five region deletions (blue, green, and brown). Also shown are the significances, calculated as Kruskall–Wallis tests with Dunn’s correction, of the difference in mobility between pairs of mutants. *ns* indicates no significant difference, two asterisks indicate p-value < 0.01, and four asterisks indicate p-value < 0.0001. *n* is between 15 and 20 granules per condition. Source data underlying graphs are provided in Source Data. **d** conservation of the PopZ protein regions. Graphical representation of a multiple alignment of 99 PopZ homologs within the *Caulobacterales* order. Each row corresponds to a PopZ homolog and each column to an alignment position. All homologs encode an N-terminal region (green), an IDR (blue), and a C-terminal helical region (brown). White regions indicate alignment gaps, and gray regions indicate predicted helices 1 to 4. Phylogeny tree of the corresponding species is shown, highlighting the four major genera in the *Caulobacterales* order: *Asticcacaulis* (pink), *Brevundimonas* (gray), *Phenylobacterium* (light purple), and *Caulobacter* (dark purple). Notably, all species within the *Brevundimonas* genus code for insertion between helix 2 and helix 3.

PopZ is composed of three protein domains^29,31^ (Fig. 3a, Supplementary Fig. 3a): (i) a short N-terminal helical region used for client binding^29,36^, (ii) a 78 amino-acid (aa) IDR (IDR-78)^36^, and (iii) a helical C-terminal region which is required for PopZ self-oligomerization^31^. To uncover the molecular mechanism driving PopZ phase separation, we examined the contribution of each of these domains to its condensation in human and *Caulobacter* cells. PopZ mutants missing either the N-terminal region (Δ1–23) or the IDR (Δ24–101) were able to form condensates in both cell types (Fig. 3b) with reduced fluidity compared to full-length PopZ (Fig. 3c and Supplementary Fig. 3b, c). In *Caulobacter*, deletion of the IDR produced dense microdomains, while in human cells, this deletion resulted in the formation of irregular gel-like condensates characterized by arrested fusion events and near to complete loss of mobility (Fig. 3b, c and Supplementary Fig. 3b, c). In contrast, deleting fragments of the predicted C-terminal helical region (Δ102–132, Δ133–156, and Δ157–177) markedly reduced visible PopZ condensates and increased condensate fluidity (Fig. 3b, c and Supplementary Fig. 3b, c). We conclude that the folded C-terminal region provides sufficient multivalency to drive condensation, and the IDR tunes the emergent material properties of the resulting condensates. This mechanism resembles that of the physiological stress sensor Pab1 from *S. cerevisiae*, whose heat shock-induced condensation is driven by its folded RNA-binding domains, while a proline-rich linker regulates temperature sensitivity^11^.

**Fig. 4:**
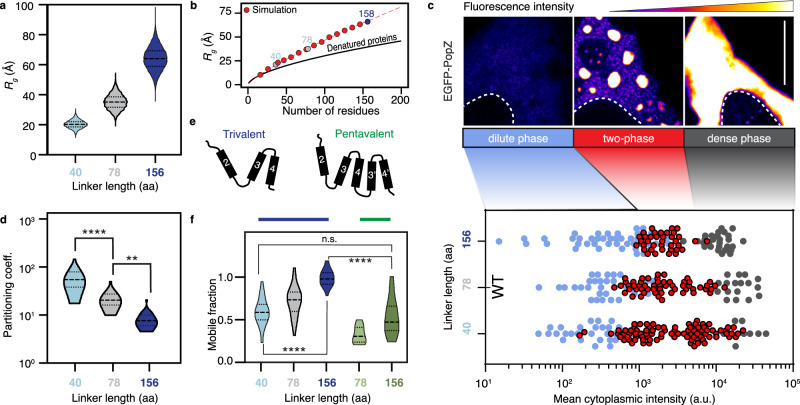
Modular organization regulates the dynamics of the PopZ condensate. **a** The predicted radius of gyration (R_G_) for a half linker (IDR-40, 40 aa) (light blue), full wildtype linker (IDR-78, 78 aa) (gray), and a double linker (IDR-156, 156 aa) (dark blue). **b** PopZ linker expands beyond the denatured limit. The expected R_G_ of denatured proteins as a function of the number of amino acids is shown in black^91^. Dimensions of PopZ-like linkers with varying lengths are shown in red, and dimensions of IDR-40, 78, and 156 are shown in shades of blue and gray. The red dashed line is an analytical fit with a scaling value of 0.80 with a prefactor value of 1.14. **c** Phase diagram of PopZ expressed in U2OS cells. (top) Three states of PopZ condensation: dilute phase (blue, left), two-phase (a diffused phase and condensed phase, red, middle), and a dense phase (gray, right). EGFP fluorescence intensity from blue (low) to white (high) and nucleus boundary as a white dotted line. Scale bar, 10 μm. (bottom) Phase diagrams of EGFP fused to either of the three PopZ variants. Each dot represents data from a single cell, positioned on the x-axis as a function of the cell mean cytoplasmic intensity. Dot color indicates phase. **d** Quantification of the partition coefficient of each of the three linkers. A higher partition coefficient indicates denser condensates. Two-sided student’s t-test; Four (two) asterisks indicate *p*-value < 0.0001 (0.01). *n* equals 30 granules per condition. Source data underlying graphs are provided in Source Data. **e** Schematics of the oligomerization domain (OD) of the wildtype PopZ (trivalent, left) and an OD with increased valency consisting of five helices, with a repeat of helices 3 and 4 (pentavalent, right). **f** The balance between condensation promoting and counteracting phase separation tunes condensate material properties. FRAP, shown as mobile fractions, for PopZ with a trivalent OD and a linker of three different lengths (gray and blue), and PopZ with a pentavalent OD with IDR-78 (light green) and IDR-156 (dark green). Two-sided student’s *t*-test; ****p-value < 0.0001. *n* equals 25 granules for each mutant. Source data underlying graphs are provided in Source Data.

The architecture of the PopZ protein from *Caulobacter crescentus* is conserved not only within the *Caulobacterales* order (Fig. 3d), but across all α-proteobacteria (Supplementary Fig. 4a). Despite showing little sequence conservation, the IDR length exhibits a narrow distribution in *Caulobacterales* with a mean of 93 ± 1 aa, while other clades of α-proteobacteria occupy different length distributions (Supplementary Fig. 4b). To characterize the molecular behavior of the PopZ IDR, we performed all-atom simulations. We found that the IDR adopts an extended conformation, with a radius of gyration (R_G_) of 32.4 ± 4.8 Å and an apparent scaling exponent (ν^app^) of 0.72 (Fig. 4a, Supplementary Tables 1-2). Consistent with previous studies^37–40^, the strong negative charge of the PopZ IDR makes it behave as a self-repulsing polyelectrolyte, driving expansion beyond the denatured limit and tightly coupling its length to its global dimensions (Fig. 4b).

For any phase separating protein, condensates emerge when the protein concentration exceeds the saturation concentration (*c*_*sat*_). At a total protein concentration below the *c*_*sat*_, the protein is uniformly dispersed (dilute phase). When protein concentration exceeds *c*_*sat*_, demixing leads to the formation of coexisting dense and a dilute phase (two-phase regime). As the total protein concentration increases and exceeds a second threshold (*c*_*D*_), the system can shift to the dense phase regime characterized by a single large droplet that occupies the intracellular space^4,34,41^. We found that in human cells, PopZ can exist in any of these three regimes as a function of its cytoplasmic concentration (Fig. 4c), allowing us to map its full phase diagram in cells.

To test the effect of altering IDR length on c_*sat*_, we transiently transfected U2OS cells with PopZ mutants containing either a truncated or extended IDR: IDR-40, corresponding to the N-terminal half the wildtype IDR, and IDR-156, corresponding to concatenation of two wildtype IDRs (Supplementary Data 1). We then tested the ability of these variants to form condensates in human cells. First, we mapped an EGFP-PopZ phase diagram as a function of concentration and IDR length. Halving the PopZ IDR length (IDR-40) decreased *c*_*sat*_ and increased the *c*_*D*_ compared to wildtype PopZ. In contrast, doubling the PopZ IDR length (IDR-156) increased *c*_*sat*_ and decreased *c*_*D*_, resulting in a narrower two-phase window (Fig. 4c). Finally, increasing the IDR length decreased PopZ partitioning, i.e., the ratio of the total concentration in the condensed phase to that in the protein-dilute phase, (Fig. 4d) and increased FRAP dynamics (Fig. 4f) in human cells. Collectively, our data suggest that the PopZ phase diagram and the emergent material properties of its condensates are tuned by its IDR length.

Because IDR length offers one means of tuning PopZ material properties, we asked if altering the degree of multivalency could be used as an orthogonal control parameter. We increased the valency of the C-terminal helical region from three predicted helical fragments (trivalent) to five (pentavalent) by repeating the last highly conserved helix-turn-helix motif (Figs. 3d and 4e). We found that pentavalent PopZ condensates had strongly reduced FRAP dynamics compared to wildtype trivalent PopZ and a morphology reminiscent of gelation (i.e., arrested fusion events, Supplementary Fig. 5). However, by creating a double mutant where we combine the pentavalent oligomerization domain (OD) with the long IDR-156, we normalized the FRAP dynamics to a physiological range and generated condensates that were able to continue fusing together (Fig. 4f, Supplementary Fig. 5). Taken together, our work reveals a modular design with two independent functional regions (IDR, OD) through which the material properties of the PopZ condensate can be tuned, providing robust design principles for synthetic engineering of customizable condensates.

### Conserved IDR features tune PopZ material properties and modulate cell division

**Fig. 5:**
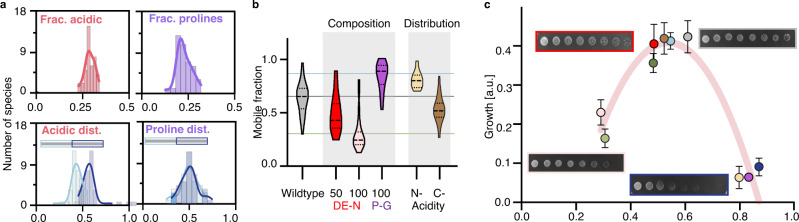
PopZ material properties are directly linked to *Caulobacter* viability and are modulated by conserved IDR properties. **a** The sequence composition of the PopZ IDR is conserved across *Caulobacterales*. Histograms are calculated across 99 PopZ homologs within the *Caulobacterales* order and show a tight distribution for the following four parameters. (top, left) The mean fraction of acidic residues is 0.29 ± 0.004 (red). (top, right) The mean fraction of prolines is 0.23 ± 0.006 (purple). (bottom, left) Among the acidic residues within the IDR, the fraction of those found in the N-terminal half (light blue, 0.57 ± 0.011) and the C-terminal half of the IDR (dark blue, 0.43 ± 0.011). (bottom, right) Among the IDR prolines, the fraction of those found in the N-terminal half (light blue, 0.5 ± 0.015) and the C-terminal half of the IDR (dark blue, 0.5 ± 0.015). Source data underlying graphs are provided in Source Data. **b** Amino acid composition plays a role in PopZ mobility. FRAP, shown as mobile fractions, for PopZ with its wildtype IDR (light gray) and five mutants: Substituting either half or all of the acidic residues for asparagine (DE-N 50% in red and DE-N 100% in pink, respectively), substituting all prolines for glycines (P-G 100% in purple), and moving all acidic residues to either the N-terminal part or the C-terminal part of the linker (yellow and brown, respectively). *n* equals 20 granules per condition. **c** Growth is linked to PopZ’s material state. Growth, derived from serial dilution growth assay (Methods), as a function of FRAP mobility for ten mutants. These include from left to right: 100% DE-N (pink), Pentavalent (light green), 50% DE-N (red), IDR-156+pentavalent (green), C-acidity (orange), IDR-40 (light blue), wildtype (gray), 100% P-G (purple), N-acidity (yellow), and IDR-156 (blue). Examples of serial dilutions are shown for wildtype (gray box), 50% DE-N (red box), IDR-156 (blue box), and 100% DE-N (pink box). A polynomial fit with an R-square of 0.86 is shown in red. Three biological replicates, each with three technical replicates, were measured for each strain. a.u., arbitrary unit.

In addition to its conserved length (Supplementary Fig. 4b), the PopZ IDR shows conservation of its strong enrichment for acidic and proline residues across *Caulobacterales*, with a − 0.28 net charge per residue and prolines constituting 29% of the IDR residues (Fig. 5a). To test whether amino acid content plays a role in the emergent properties of the PopZ microdomain, we substituted acidic residues for asparagine and proline residues for glycine. Decreasing the negative charge of the linker reduced condensate mobility in human cells while substituting prolines for glycines slightly increased condensate mobility (Fig. 5b). In addition to amino acid composition, the PopZ IDR shows conservation of charge patterning (Fig. 5a)^42^. We tested an array of IDR-scrambled mutants and found that the distribution of negative charge along the primary sequence modulates material properties (Supplementary Fig. 6). Further, our all-atom simulations suggest that charge distribution may modulate interdomain interactions (Supplementary Fig. 7). Thus, the charged PopZ IDR tunes material properties in a predictable manner, based on specific sequence properties that show strong conservation across *Caulobacterales* despite differences in the primary IDR sequence. This strong conservation of IDR length, amino acid composition, and primary sequence features that tune intra-condensate PopZ dynamics suggests that the microdomain material state may be important for its biological function.

We next expressed the different PopZ IDR mutants in Δ*popZ Caulobacter* cells and found that FRAP dynamics were consistent between *Caulobacter* and human cells (Supplementary Fig. 8a, b). Assaying *Caulobacter* growth in PopZ mutant strains revealed that optimal fitness was achieved by wildtype PopZ. Mutants that form condensates that are either too solid or too fluid exhibited reduced fitness (Fig. 5c). This is particularly notable given that the trivalent OD, linker length, proline content, and acidity content are conserved (Figs. 3d and 5a). Importantly, both the pentavalent mutant, resulting in solid-like condensates, and the long IDR-156 mutant, resulting in fluid condensates, lead to reduced fitness. Yet, by combining these two independent loss-of-function mutants into a double mutant, we restored wildtype material properties and fitness (Fig. 5c). Collectively, we identified a narrow range of material properties, a ‘Goldilocks’ zone, where the PopZ microdomain is fully functional and properly orchestrates cell division.

### PopZ material properties alter microdomain localization and client recruitment

**Fig. 6:**
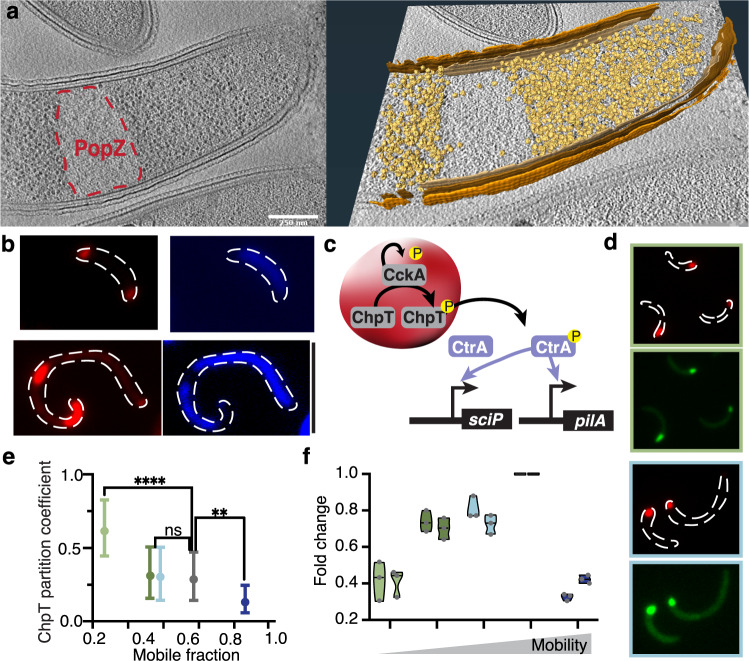
PopZ material properties affect cytosol organization. **a** PopZ IDR-156 condensates retain ribosome exclusion. (left) Slice through a tomogram of a cryo-focused ion beam-thinned Δ*popZ Caulobacter* cell overexpressing mCherry-PopZ with IDR-156. (right) Segmentation of the tomogram in (left) showing annotated S-layer (orange), outer membrane (dark brown), inner membrane (light brown), and ribosomes (gold). Scale bar, 0.25 μm. **b** PopZ IDR-156 condensates retain DNA exclusion. PopZ IDR-156 condensates expressed in ΔPopZ cells dynamically moved in the cytosol and excluded DAPI-stained DNA (blue). Scale bar, 5 μm. **c** The CtrA activation network is sequestered to the PopZ condensate. The schematic shows the auto-kinase CckA^92^ phosphorylating the phospho-transfer protein, ChpT^45^, which in turn phosphorylates the master transcription factor, CtrA^93^. All three proteins are sequestered to the PopZ condensate^26,29,30^. Phosphorylation of CtrA occurs largely inside the condensate^30^. CtrA~P leaves the DNA-free PopZ condensate and activates an array of asymmetry regulating genes, including *sciP*^94,95^ and *pilA*^96^. **d**, **e**. PopZ material properties affect ChpT recruitment. **d** Representative cells are shown for pentavalent, IDR-40, and IDR-156. **e** The graph shows the partition coefficient of ChpT inside PopZ condensates as a function of condensate mobility. The coefficient was calculated as ChpT fluorescence intensity inside the PopZ condensates divided by the fluorescence intensity outside the condensates. A higher partitioning coefficient indicates stronger recruitment. Data shown for pentavalent (light green), pentavalent with IDR-156 (green), IDR-40 (light blue), wildtype (gray), and IDR-156 (blue). *n* equals 60 cells per strain. Two-sided student’s *t*-test; *ns* indicates no significant difference, two asterisks indicate *p*-value < 0.01, and four asterisks indicate p-value < 0.0001. Source data underlying graphs are provided in Source Data. **f** PopZ material properties affect the transcriptional program regulating asymmetry. Expression levels of CtrA activated genes *sciP* and *pilA* in cells expressing different PopZ mutants. Color code as in **e**. Three biological replicates (gray points), each with at least two technical replicates, were measured for each strain and each gene. Source data underlying graphs are provided in Source Data.

PopZ localization to the bacterial cell poles is attributed to nucleoid exclusion^43^. In *Caulobacter*, the nucleoid spreads through most of the cytosol, restricting PopZ to the DNA-free cell poles. Here, we asked whether the material properties of PopZ condensates influence their polar localization. Both solid and liquid PopZ condensates retain their ability to form a barrier that excludes ribosomes and DNA, as measured by correlative cryo-electron tomography (Fig. 6a, Supplementary Fig. 9) and DAPI staining (Fig. 6b, Supplementary Fig. 10a), respectively

In wildtype *Caulobacter* cells, the PopZ microdomain is localized to the “old” cell pole during the initial stages of the cell cycle. Upon cell growth and replication, a new microdomain is established at the opposite “new” cell pole^25,26^. This behavior changed when replacing wildtype PopZ with mutants that led to either liquid or solid PopZ condensates. On the one hand, liquid condensates formed by the IDR-156 mutant lost their polar localization and diffused throughout the cell (Supplementary Fig. 8a, e), leading to a constant reorganization of ribosome distribution and DNA structure (Supplementary Figs. 9 and 10a). Given that this mutant could not retain polar localization, it effectively prevents the PopZ microdomain from establishing asymmetry, thereby perturbing its control over asymmetric cell division and explaining its loss-of-function phenotype. On the other hand, solid condensates, formed by the pentavalent mutant, were able to reliably localize to one cell pole but did not form a second microdomain at the opposing (“new”) cell pole and therefore were also unable to reliably complete cell division (Supplementary Fig. 8c, d).

The PopZ microdomain acts as a signaling hub by recruiting members of the phospho-relay pathway that activates the CtrA transcription factor, which regulates asymmetric division^27,29,30^ (Fig. 6c). We asked whether the material properties of PopZ condensates affected the orchestration of this pathway. We first examined the ability of PopZ mutants to recruit a member of the phospho-relay pathway. ChpT directly binds both CtrA^44^ and PopZ^30^ and activates CtrA by phosphorylation^45^. ChpT recruitment was increased in solid PopZ condensates and reduced in liquid condensates (Fig. 6d, e, Supplementary Fig. 10b), indicating that correct ChpT partitioning is a function of PopZ material properties. We then assayed the effect of the PopZ material state on CtrA activation by measuring the expression level of the CtrA-regulated genes *pilA* and *sciP*. The expression of both genes was dependent on the material properties of the PopZ condensate–too solid or too fluid were correlated with improper activation of their transcription (Fig. 6f).

Collectively, our data reveal that too solid-like or too fluid-like microdomains interfere with their proper subcellular localization, alter client recruitment, and ultimately deregulate the signaling pathways driving asymmetry and cell division. Thus, we suggest that the function of the PopZ microdomain is tightly linked to its material properties, which have been precisely tuned to meet the cell’s needs. Given the importance of bipolar localization of PopZ microdomains to the progression of the *Caulobacter* cell cycle, we speculate that cells’ inability to properly localize too solid and too liquid condensates underlies, in part, their non-functionality. As the valency of the OD can restore IDR length phenotypes and vice versa (Fig. 4f), we suggest that a tight balance of opposing forces mediated by the IDR and the OD define this physiological window. Indeed, analyzing sequences of PopZ homologs, we found that increased linker length is compensated for by a reduction in the charge fraction (Supplementary Fig. 11a). Therefore, the PopZ IDR sequence might be tuned in natural populations to constrain deviations in material properties to ensure proper cell division (Supplementary Fig. 11b).

### Material properties tune features of synthetic condensates

**Fig. 7:**
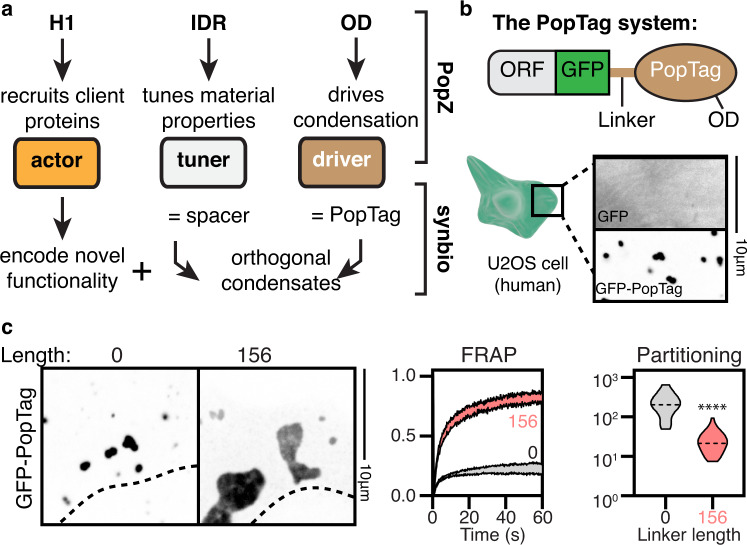
A modular platform for generating synthetic condensates with tunable properties. **a** Re-engineering PopZ as a modular platform for the generation of designer condensates. The oligomerization domain (PopTag) drives phase separation, the IDR (spacer) tunes material properties, and the n-terminal domain (actor) determines functionality. **b** Scheme highlighting setup of the PopTag system and formation of EGFP-PopTag condensates in U2OS cells. **c** Changing the linker length alters the FRAP dynamics and partitioning coefficient of PopTag condensates. Two-sided student’s t-test; ****p-value < 0.0001. For the FRAP dynamics plot, 15 condensates were analyzed per condition, and 30 condensates were analyzed per condition for the partitioning coefficient plot. Source data underlying graphs are provided in Source Data.

**Fig. 8:**
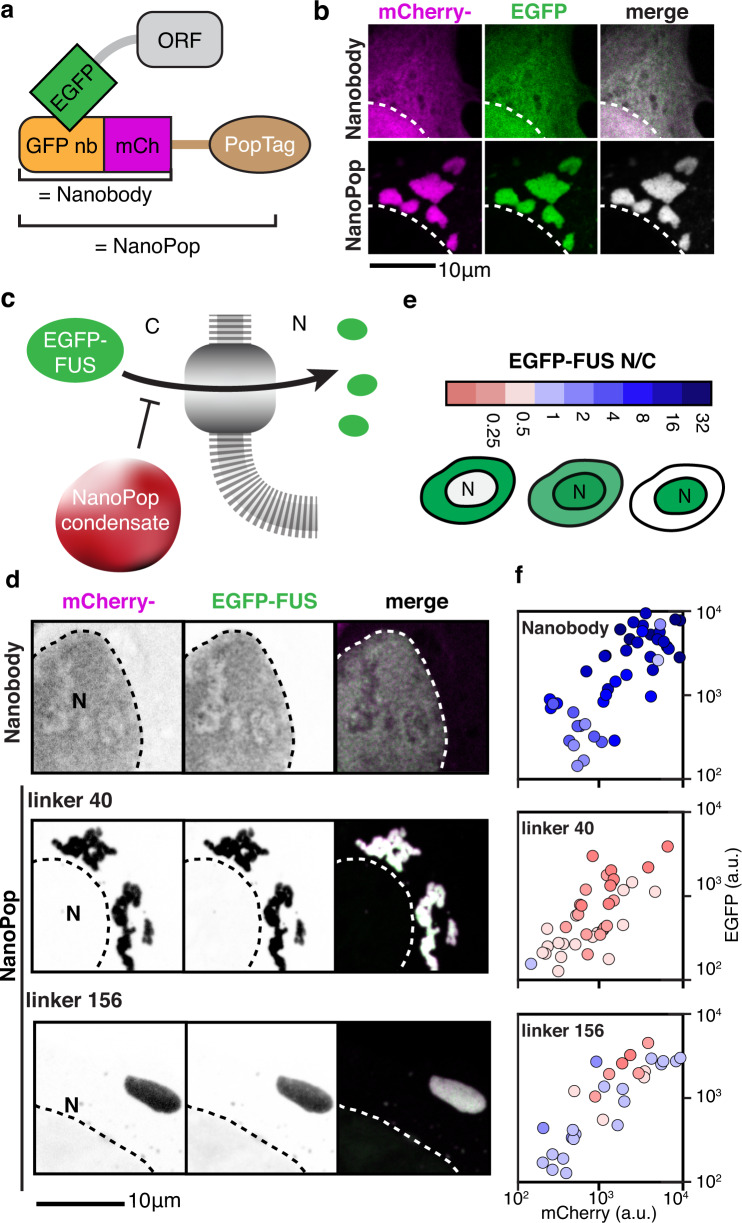
NanoPop can inhibit nuclear import. **a**, **b** The NanoPop system. **a** NanoPop is the fusion of the PopTag to a nanobody (nb), which allows the recruitment of clients into cytoplasmic condensates. In this example, PopTag is fused to a GFP nb, which allows the recruitment of EGFP-tagged protein. (**b**, top) Cells expressing EGFP and GFP nb fused to mCherry (GFPnb-mCherry) show diffused EGFP, diffused GFPnb-mCherry, and no correlation between them. (**b**, bottom) Cells expressing EGFP and GFPnb-mCherry-PopTag show GFPnb-mCherry-PopTag condensates with co-localized EGFP. **c** Schematics of the NanoPop system with EGFP fused to FUS. **d**–**f** NanoPop can inhibit nuclear import of FUS. N = nucleus, C = cytoplasm. **d** Co-expression of an EGFP-targeting nb does not impair the nuclear import of EGFP-FUS, whereas co-expression of NanoPop does. The strength of this effect is dependent on the NanoPop linker length (PopZ linker 40 versus PopZ linker 156). **e** Nuclear import is quantified by the nucleocytoplasmic ratios (N/C), the EGFP-FUS signal in the nucleus divided by the signal in the cytoplasm. Schematics of EGFP signals for low, medium, and high N/C are shown. **f** Quantification of EGFP-FUS N/C dependence on the material properties of its recruiting protein. Axes indicate average cellular mCherry and EGFP intensity for co-expression of EGFP-targeting nb alone (top), NanoPop-L40 (middle), and NanoPop-L156 (bottom). The color code indicates N/C, as illustrated in **e**. *n* equals 45, 39, and 31 cells for Nanobody, NanoPop-L40, and NanoPop-L156, respectively. a.u., arbitrary unit.

The simple modular domain architecture of PopZ provides a tunable platform for generating designer condensates^46^ (Fig. 7a). This architecture includes a C-terminal oligomerization domain (OD) that drives condensation, an IDR that tunes its material properties, and an “actor” N-terminal client binding domain. We found that the PopZ OD (which we named PopTag) was sufficient to drive cytosolic condensate formation in human cells (Fig. 7b). The material properties of these PopTag condensates could be further tuned by the addition of a PopZ IDR variant (spacer, Fig. 7c), consistent with our finding on IDR effect on mobility for full PopZ condensates (Figs. 4a, c and 5c). We then constructed NanoPop, a fusion between PopTag and a GFP-targeting nanobody (Fig. 8a). The NanoPop condensates efficiently sequestered EGFP into cytoplasmic condensates (Fig. 8b).

In light of our observation that the material properties of PopZ condensates modulate their ability to recruit clients (Fig. 6d, e), we asked whether the material properties of the synthetic NanoPop constructs would affect their recruitment ability as well. We turned to the nuclear RNA-binding protein FUS which is reversibly sequestered to cytoplasmic physiological liquid-like stress granules^47^ (Fig. 8c). In certain neurodegenerative diseases, amyotrophic lateral sclerosis and frontotemporal dementia, FUS irreversibly condenses into solid-like cytoplasmic aggregates, suggesting a connection between material properties and the extent of nuclear depletion. Accordingly, we examined the effect of the material properties of NanoPop condensates on the recruitment efficiency of EGFP-FUS. Nuclear import of EGFP-FUS was not hindered by co-expressing the GFP nanobody (Fig. 8d–f). Co-expression of NanoPop led to the sequestration of EGFP-FUS in the cytoplasm NanoPop condensates. Notably, the extent of sequestration depended on the spacer length of NanoPop (Fig. 8d–f). NanoPop with an IDR-40 resulted in small solid-like condensates that displayed arrested fusion and depleted FUS from the nucleus (i.e., nucleocytoplasmic ratio < 1). NanoPop with an IDR-156 resulted in larger, more spherical condensates that were less efficient at sequestering FUS from the nucleus. We found a 2 to 4 folds reduction in EGFP-FUS recruitment into L156 NanoPop compared to L40 NanoPop at equal expression levels. Collectively, we found that recruitment of EGFP-FUS was most efficient for solid-like NanoPop condensates, similar to our observation regarding ChpT recruitment by PopZ condensates (Fig. 6d, e). Thus, altered material properties of condensates can partly explain their pathological features and highlight the importance of proper condensate regulation for cell physiology.

We further show that PopTag can be used to make a wide array of designer condensates by fusing it to different actor domains. This allows one to create orthogonal condensates with tunable localization, such as localization to actin filaments and lipid droplets (Supplementary Fig. 12a, b), tunable clients such as enzymes (Supplementary Fig. 12c), as well as tunable stability using a chemically induced degradation^48^ (Supplementary Fig. 12d). Therefore, we anticipate that the PopTag platform will provide a way to decipher the contributions of specific condensate features to their role in physiology and pathogenesis and constitutes a versatile tool for synthetic biology applications.

## Discussion

Intrinsically disordered proteins are estimated to make up 4% of bacterial proteomes, unlike 30–50% of eukaryotic proteomes^49^, perhaps explaining why their role in bacterial physiology has been largely overlooked. Accumulating evidence suggests that these proteins play vital roles in bacterial cell physiology, including in the biogenesis of a growing number of bacterial biomolecular condensates^50,51^. Biomolecular condensates occur across the tree of life^52,53^ and are involved in multiple cellular processes^2,54^. What sets condensates apart from their membrane-bound counterparts are their emergent properties, which refer to the material properties that emerge once a protein switches from a mixed to a condensed state. Thus, a key question is whether these condensates are important for protein function. Indeed, a growing list of studies has shown that condensation of a protein complex is important for its function^11–16^. It remains less clear whether the exact material properties contribute to function. Several condensate proteins have been implicated in human disease^55,56^ and have been reported to form pathological amyloid aggregates in patients^9,18–21,57^. While this has been suggested as evidence that condensate material properties are important for their biological function, these proteins usually aggregate outside the context of their physiological condensate (e.g., TDP-43 aggregates outside of stress granules^58,59^). Other studies have shown that affecting the liquidity of condensates can alter their function^22,23^, yet the effect of condensate fluidity, from solid to liquid, on condensate function and organismal fitness is largely underdetermined. Here, we used the PopZ microdomain as a case study to explore the relationship between biophysical state and biological function.

The intrinsically disordered protein PopZ forms microdomains at the cell pole of the bacterium *Caulobacter crescentus*. Previously, we have shown that these membraneless assemblies selectively recruit kinase-signaling cascades to regulate asymmetric cell division^25,26,30^. We now report that PopZ forms these assemblies via phase separation and provide evidence that it is the necessary and sufficient condensate scaffold (Figs. 1 and 2). Next, we dissected the molecular grammar of PopZ, revealing a push-pull mechanism mediated by a helical OD that drives condensation and a repulsive IDR that fluidizes the assembly (Figs. 3 and 4). These two domains act as independent tuning knobs of the PopZ material state. On the one hand, modulating the valency of the OD alters the condensate material state. Specifically, increasing the number of OD helices from three to five strongly promoted condensation (Fig. 4e, f) while decreasing the number of helices from three to two weakened condensations (Fig. 3b, c). On the other hand, modulating the expanded and repulsive nature of the IDR promotes or decreases PopZ phase separation and tunes its material properties (Figs. 4d, e and 5b, Supplementary Fig. 6).

Using bioinformatics analysis, we have identified conserved IDR characteristics, despite a lack of primary sequence conservation, that are important in tuning PopZ mobility, suggesting that its material state may be under selective pressure (Fig. 5, Supplementary Fig. 4). If these conserved IDR features resulted from selective pressure, one would predict that modulating these features would alter the precise balance of attractive and repulsive forces, thereby perturbing PopZ material properties and its biological function. Indeed, by testing rationally designed mutants spanning the material properties spectrum, we show that there exists a Goldilocks zone of material properties where PopZ is functional. Deviations from this optimum, either too liquid or too solid, perturb proper cell division and decrease fitness (Figs. 5 and 6, Supplementary Figs. 8–10). Specifically, we found that altered material states result in the subcellular mislocalization of the PopZ microdomain (Supplementary Fig. 8), affect client recruitment (Fig. 6d–e), and result in a failure to activate the correct transcriptional program to drive asymmetric cell division (Fig. 6f). These findings provide evidence for the role of condensate material properties in tuning their biological function. Future work is required to fully understand the relationship between PopZ condensate properties and *Caulobacter* cell cycle regulation.

Cells have evolved several strategies to compartmentalize their biochemical reactions to manage their complexity. Stoichiometric protein machines (e.g., enzyme complexes) execute multiple cellular functions^60^. The cytoskeleton allows for rapid and directed transport of RNA or vesicles^61^, and intracellular membranes form specialized organelles^62^. Phase separation is emerging as another ubiquitous organizing principle that is critical for many biological processes in all cells, from bacteria to humans. What sets condensates apart from these other organizational mechanisms are their emergent properties. Hence, if evolution had selected for compartmentalization through phase separation, one would expect that there should indeed exist a limited range of material properties that correspond to biological function. In the case of PopZ from the aquatic bacterium *Caulobacter crescentus*, we indeed find it to be true. Examining PopZ across α-proteobacteria, we found that while the OD sequence is highly conserved, the IDR sequence changes its conserved features across clades (Supplementary Fig. 4). The divergent sequence of IDRs provides a mechanism for natural selection to tailor a condensate’s material properties to a particular environmental niche. While speculative at this point, we anticipate that study of condensates in bacteria could reveal strategies of condensate adaptation.

Lastly, inspired by the simple modular domain architecture of PopZ (Fig. 7a), we developed a synthetic biology platform for the generation of designer condensates^46,63,64^ (Fig. 7b, c) with a variety of functionalities and tunable properties (Fig. 8, Supplementary Fig. 12). The synthetic constructs presented in this work were designed to showcase the versatility of the PopTag system, here transiently expressed in U2OS cells. Future work will be aimed at testing the utility of PopTag across expression levels, cell lines, and model organisms. In this context, PopTag was used to study how short linear motifs can recruit specific client proteins into condensates and to elucidate the behavior of condensate modifiers^65^. Finally, since bacterial IDRs differ from their eukaryotic counterparts, not only in proteome abundance but also in amino acid composition^49,66^ (Supplementary Fig. 1c), we imagine that further exploration of the prokaryote sequence space may provide us with additional tools to engineer orthogonal biomolecular condensates for eukaryotic cells.

## Methods

### PopZ purification

PopZ was expressed and purified from *E. coli* strain BL21. Cells were grown to an OD of 0.4 and then switched to induction temperatures of 30 °C for 30 min prior to induction. PopZ expression was induced with 1 mM IPTG for 2 h. Cell pellets were collected via centrifugation and stored at −80 °C.

PopZ was purified under denaturing conditions^31^. The frozen cell pellet was resuspended in lysis buffer containing 100 mM Na-phosphate, 10 mM Tris-Cl pH 8.0, 300 mM NaCl, 8 M urea, 20 mM imidazole, and 1 tablet of EDTA-free UltraCruz protease inhibitors (Santa Cruz Biotechnology) for each 50 mL of lysis buffer. A total of 1.2 g of guanidinium chloride was added per 1 L cell culture, and the cells were homogenized by passage through a 21-gauge needle. Insoluble material was removed via centrifugation at 12,100 g for 45 min at room temperature. The supernatant was incubated for 2 h with 1 mL His-Pur Ni-NTA agarose resin (ThermoFisher) per 1 L of initial cell culture. The resin was washed 4 times in lysis buffer. The protein was eluted using 4 mL elution buffer (lysis buffer modified to have 250 mM imidazole) per 1 L initial culture. The PopZ protein was concentrated using a 30 k MWCO spin filter (Millipore). The protein was refolded during 3 rounds of dialysis at 4 °C in 20 mM Tris-Cl buffer at pH 8.0.

### Turbidity assay

The turbidity measurements at 350 nm were performed using a NanoDrop 2000c UV–vis spectrophotometer at room temperature. Buffer, PopZ, and MgCl_2_ were all in solutions at pH 6.0 and in the presence of 5 mM sodium phosphate. For each condition, reagents were mixed to yield a final volume of 60 µL and incubated for 5 min in a 10 mm path length quartz cuvette (26.10LHS-Q-10/Z8.5, StarnaCells). The cutoff value of A_350nm_ = 0.02 was determined by whether or not droplets were visible after 5 to 20 min of incubation under a light microscope with a 100× oil immersion objective (Plan-Neofluar, 100×/1.30 Oil Pol M27).

### Differential interference contrast

DIC imaging was performed on a Zeiss LSM 880 laser scanning confocal microscope, equipped with a 63× oil immersion objective (Plan-Apochromat 63×/1.4 Oil DIC M27). For each condition, a 20 µL sample was prepared, incubated for 10 min, and imaged using a Nunc Lab-Tek Chambered Cover-glass (ThermoFisher Scientific Inc) at room temperature.

### Construction of plasmids and strains for *Caulobacter* studies

Plasmids, strains, and primers are listed in Supplementary Tables 3-5.

#### Plasmids

AP211 (pBXMCS-2 mCherry-PopZ) was amplified with primer pair 1 to remove the PopZ IDR. IDR-40 was synthesized as a gBlock gene fragment (IDT) and inserted into the linearized AP211 by Gibson assembly^67^ to make pKL539. pKL540 and pKL577 were constructed similarly with primer pairs 2 and 3 and gBlock gene fragments that codes for IDR-156 and H3-H4, respectively. To make pKL581, pKL540 was amplified with primer pair 3, and gBlock H3-H4 was inserted into the linearized pKL540 by Gibson assembly. To make pKL699-704, AP211 was digested with KpnI and SacI. Corresponding gBlocks were inserted into the digested and linearized AP211 by Gibson assembly. PCRs were performed with the KOD Hot-start 2× master mix (Novagen), and cloning was performed using Gibson Assembly 2× Master Mix (New England BioLabs, NEB) following the manufacturer’s instructions. The sequence of each insert was verified by Sanger sequencing (Sequetech).

#### Strains

To make KL6212, purified plasmid pBXMCS-2 mCherry-PopZ from AP211 cells was electroporated into KL5943. To make all other strains, purified plasmids were transformed into Δ*popZ* cells by electroporation and plated on marked PYE plates. The resulting colonies were screened for mCherry fluorescence after induction with xylose and confirmed by western blots.

### Imaging *Caulobacter* cells

Images were collected using a Leica DMi8 S microscope equipped with a Hamamatsu C9100 EM‐CCD camera, a 100× oil-immersion objective (1.63 NA), and a SPECTRA X light engine (Lumencor). Cell outlines and intensity profiles were identified using MicrobeJ^68^ and manually filtered to eliminate false positives. Custom MATLAB 2020a (The MathWorks) scripts were used to calculate the average fluorescence intensity profile along the long axis of the cell.

### FRAP measurements in *Caulobacter* cells

Photobleaching experiments were performed using an LSM710 line-scanning confocal microscope (Zeiss) with a 60× oil immersion objective with a numerical aperture (NA) equal to 1.4. A circular region of interest (ROI) within a PopZ microdomain was bleached using a high-intensity 561 nm laser and 50% bleaching power. Pictures at a rate of five per minute were taken for 3 min. Control pictures (cells and background) were taken under the same conditions. Normalization and photobleaching corrections were performed^17^.

### Serial dilution plating viability assay

Strains were grown in M2G with appropriate antibiotics to an OD600 of 0.3. Ten microliters of each dilution were spotted onto PYE plates in triplicates. Plates were incubated at 30 °C for two days and imaged with Gel Doc XR Imaging System (BioRad). The mean density for each spot was calculated using lmageJ/FIJI version 1.53c following background subtraction. The growth value for each strain was defined as the mean density at the sixth dilution divided by the mean density at the first dilution. Parabolic fit was conducted using GraphPad Prism 9.3.1.

### RT–qPCR gene expression

The effects of PopZ material properties on the transcription rate of the *sciP and pilA* genes were determined by measuring mRNA levels with RT–qPCR. RNA was extracted using Monarch Total RNA Miniprep Kit (cat#T2010S, NEB). In-tube DNase I treatment was performed following RNA extraction to eliminate residual genomic DNA. The RNA was subsequently reverse-transcribed using the SuperScript III First-Strand Synthesis System (cat#18080051, Invitrogen). Following reverse transcription, the remaining RNA was degraded via RNase H treatment, and the complementary DNA (cDNA) was diluted tenfold before beginning qPCR.

Expression levels were determined using a CFX Connect Real-Time PCR system (Bio-Rad), using the Bio-Rad CFX Manager 3.1 software. The 15 µL qPCR reaction contained 2 µL of cDNA, 7.5 µL of SYBR Green (Fast SYBR Green master mix, cat#4385612, Applied Biosystems), and 5.5 µL of primer mix. The primer mix contained the forward and reverse primers to form approximately 100-bp amplicons in the genes of interest at a final primer concentration in the reaction of 230 nM. Expression measurements were then made by comparing the cycle threshold (C_T_) of the amplicons of interest to an internal standard amplicon in *rho*. This housekeeping gene is insensitive to cellular concentrations or activity of CtrA. As a negative control, we verified the removal of gDNA template contamination by measuring the C_T_ of RNA samples not treated with reverse transcriptase. We additionally measured the amplification of a gDNA standard curve to verify that the Rho, SciP, and PilA amplicons formed unique products and with amplification efficiencies within 10% of one another; we measured amplification efficiencies of 102%, 101%, and 91%, respectively. Data were analyzed using the ∆∆C_T_^69^ method. Final gene expression measurements represent the average and SEM of three biological replicates, each composed of at least two technical replicates.

### Bioinformatics

PopZ homologs were identified based on the C-term region using BLAST^70^. Taxonomy was extracted from NCBI. A phylogenetic tree was determined based on the full-length sequence using Geneious Prime 2020.0.4 (https://www.geneious.com). NetSurfP-2.0^71^ was used to detect intrinsically disordered regions, and JPred^72^ to detect secondary structures in the full-length homologs. Custom scripts written in python 3.7.3 were used for regression analysis and visualization.

### All-atom simulations

All-atom simulations were run using the ABSINTH abs3.2_opls.prm implicit solvent model and the CAMPARI V2 Monte Carlo simulation (http://campari.sourceforge.net/)^73^. The combination of ABSINTH and CAMPARI has been used previously to effectively sample the conformational behavior of disordered proteins with good agreement to experiment, notably in the context of highly charged and highly proline-rich IDRs^37,74^. All simulations were started from randomly generated non-overlapping random-coil conformations, with each replica using a unique starting structure. Monte Carlo simulations evolve the system via a series of moves that perturb backbone and sidechain dihedral angles along with the rigid-body coordinates of both polypeptides and explicit ions. Simulation analysis was performed using SOURSOP 0.1.9 (https://soursop.readthedocs.io/) and MDTraj 1.9.5^75^ (http://mdtraj.org). The protein secondary structure was assessed using the DSSP algorithm^76^.

ABSINTH simulations were performed with the ion parameters derived by Mao et al., with the notable exception of the double linker for which an enhanced Na^+^ ionic radius (2.32 Å vs. 1.16 Å) was applied to prevent non-physiological chelation^77^. All simulations were run at 10 mM NaCl and 310 K. An overview of the simulation input details is provided in Supplementary Table 1. A summary of simulation analysis statistics is provided in Supplementary Table 2.

A major challenge in the sampling of disordered proteins reflects an effective exploration of conformational space. The highly repulsive and expanded nature of the linker provides some advantages in that conformational space is substantially reduced by the polyelectrolytic nature of the chain. Simulations reveal no substantial secondary structure (Supplementary Fig. 13a), with good agreement between analogous sub-regions examined in different length constructs. Further, histograms of Rg revealed a smooth distribution consistent with a well-sampled ensemble without substantial local kinetic traps (Supplementary Fig. 13c). To assess sampling for full-length PopZ, we compared simulation-derived secondary structure profiles for wildtype PopZ, N-acidity, and C-acidity mutants (Supplementary Fig. 13b). In agreement with good conformational sampling, we observed nearly perfectly overlapping helicity profiles for the N and terminal regions that remain unchanged between the three constructs, giving us confidence that simulations are relatively converged with respect to the relevant order parameters of interest. As with the linker constructs, smooth distributions for the *R*_*g*_ are again consistent with a well-sampled conformational ensemble (Supplementary Fig. 13d).

### Construction of plasmids for human cell lines studies

PopZ and derived mutant constructs for expression in human cells were generated through custom synthesis and subcloning into the pcDNA3.1 + N-eGFP backbone by Genscript (Piscataway, USA). The mCherry-G3BP1 plasmid was a kind gift from Dr. Kedersha and Dr. Anderson (Brigham and Women’s Hospital). Sequences are found in Supplementary Data 2.

### Human cell culture and microscopy

U2OS cells (ATCC, HTB-96) cells were grown at 37 °C in a humidified atmosphere with 5% CO_2_ for 24 h in Dulbecco’s Modified Eagle’s Medium (DMEM), high glucose, GlutaMAX + 10% Fetal Bovine Serum (FBS) and pen/strep (ThermoFisher Scientific). Cells were transiently transfected using Lipofectamine 3000 (ThermoFisher Scientific) according to manufacturer’s instructions. Biotinylation experiments using TurboID-PopTag condensates were performed as described in detail^78^. Cells grown on coverslips were fixed for 24 h after transfection in 4% formaldehyde in PBS. Slides were mounted using ProLong Gold antifade reagent (Life Technologies). Confocal images were obtained using a Zeiss LSM 710 confocal microscope. Images were processed using FIJI.

### FRAP measurements in human cells

U2OS cells were cultured in glass-bottom dishes (Ibidi) and transfected with GFP-PopZ constructs as described above. After 24 h, GFP-PopZ condensates were bleached, and fluorescence recovery after bleaching was monitored using Zen software on a Zeiss LSM 710 confocal microscope with an incubation chamber at 37 °C and 5% CO_2_. Data were analyzed as described previously^17^. In brief, raw data were background-subtracted and normalized using Excel, part of Microsoft 365 version 2007, and plotted using GraphPad Prism 9.3.1 software.

### Statistical analysis

All data were analyzed using GraphPad Prism 9.3.1. Statistical test details are shown in figure legends.

### Cryo-electron tomography

#### Sample preparation

Log phase *Caulobacter* (OD600 between 0.2 and 0.5) grown in M2G media were diluted 1:10 in fresh M2G media and induced for 4–5 h with 3% xylose at 28 °C in a shaking incubator. For plunge freezing of *Caulobacter*, induced cells were placed on ice and concentrated to an effective OD600 of 3.0 by centrifugation. For whole-cell tomography, cells were diluted to an effective OD600 of 0.2 and plunge frozen in a similar manner.

To reduce the formation of crystalline ice, 1 µL of 50% w/v trehalose was added to 9 µl of the cell suspension immediately before plunge-freezing. A total of 4 µL of the cell suspension were added to the carbon side of a glow-discharged Cu 200 mesh R2/1 Quantifoil grid and manually blotted from the back to remove excess liquid and were plunge-frozen in an ethane/propane mixture cooled to liquid nitrogen temperatures using a custom-built manual plunger (Max Planck Institute for Biochemistry). Grids were clipped into an Autogrid support ring to facilitate downstream handling. The frozen grids were kept at liquid nitrogen temperatures for all subsequent steps.

#### Cryo-fluorescence microscopy

Frozen grids were observed with a CorrSight inverted microscope (ThermoFisher Scientific) using EC Plan-Neofluar 5×/0.16NA and EC Plan-Neofluar 40×/0.9NA air objectives (Carl Zeiss Microscopy), a 1344 × 1024 px ORCA-Flash 4.0 camera (Hamamatsu), and an Oligochrome light-source, with excitation in four different channels (405/488/561/640 nm); red (mCherry-PopZ) and green (GFP-ribosomes) were used. Data acquisition and processing were performed using MAPS 2.1 and MAPS 3.6, respectively (Thermo Fisher Scientific). After acquiring a grid map at 5× magnification, regions of interest were imaged at 40× magnification to identify cells with PopZ domains.

#### Cryo-focused ion beam (FIB) milling

Grids with *Caulobacter* were prepared using cryo-FIB milling as previously described using an Aquilos (ThermoFisher Scientific) dual-beam SEM equipped^79,80^. Briefly, areas covered with a monolayer of cells were targeted first for coarse milling with an ion beam current of 0.10–0.50 nA, followed by fine milling using 10–50 pA. Lamella width was typically 10–12 µm. Five to eight lamellae were prepared on each grid in one session, with a target thickness of ~150 nm.

#### Cryo-electron tomography

*Caulobacter* lamellae were visualized on a Titan Krios (ThermoFisher Scientific) operating at 300 kV accelerating voltage with a Gatan K2 Summit camera equipped with a Quantum energy filter. Regions of interest were determined by correlating TEM and FM images. Tilt series of Caulobacter were obtained using SerialEM v3.8b11^81,82^ using both bi-directional and dose-symmetric tilt schemes^83^ over a tilt range of ± 60°, in increments of 2° or 3°, at a pixel size of 0.4265 nm or 0.3457 nm. Each tilt image was collected using electron counting mode and with dose-fractionation. Exposure times for each tilt were adjusted to keep an approximately constant number of counts on the sensor. Cumulative dose for each tilt series was usually between 120 and 180 e/A2.

#### Tomogram reconstruction

The movies corresponding to each tilt were motion-corrected using MotionCor2 software^84^. Tilt series alignment and reconstruction were done using IMOD^85–87^. Tilt series were aligned using the patch-tracking modality and reconstructed using weighted back-projection. If needed, individual tilts with excessive motion, poor contrast, or camera errors were excluded from the final reconstruction. Non-linear anisotropic diffusion (NAD) filtering was applied to tomograms using Etomo (part of IMOD) to enhance contrast for presentation in IMOD.

#### Ribosomal template matching

Ribosome locations were determined using the template matching routine from Dynamo-v1.1.514^88^. Template matching was performed on NAD filtered, 4-binned tomograms. Briefly, a reference 70S prokaryotic ribosome (PDB: 5MDZ) was filtered to a 20 Å resolution and resampled to the appropriate pixel size to serve as the template and used in conjunction with a close-fitting spherical mask (30.7 nm or 18 binned pixels) for the template matching routine. All points located outside of the cell boundary were excluded. A cross-correlation threshold that resulted in most ribosome-like particles being included was selected, and particles above this threshold were extracted from unfiltered tomograms (2×-binned), aligned, and averaged using Dynamo. Particles residing inside the PopZ compartment (<3%) were visually inspected to verify their identity and not included if they were deemed false negative.

#### Membrane segmentation

Membranes were detected using TomoSegMemTV-vApr2020^89^. Membrane and ribosome annotations were visualized with Amira (ThermoFisher Scientific).

### Statistics and reproducibility

Phase diagrams of recombinant PopZ protein (Supplementary Fig. 1b–f) were constructed from two independent experiments with similar results. All other data were generated from at least three independent experiments with similar results. Results are expressed as means ± standard errors of the mean (SEM). For all graphs where statistical analyses were applied, the number of repeats (*n*) has been mentioned in the figure legends. No data were excluded from the analyses. GraphPad Prism-v9.3.1 was used to perform the statistical analysis. Significance is expressed as *p* values (not significant (ns), *p* > 0.5; *p < 0.05; **p < 0.01; ***p < 0.001; ****p < 0.0001). Two-sided unpaired t-test and ordinary one-way ANOVA test were used for parametric data and Kruskal-Wallis for non-parametric data.

For Figs. 1a, c, d, 6b, d, and Supplementary Figs. 8a, e, 10, images are representative examples from 3 independent biological replicates with *n* ≥ 100 *Caulobacter* cells per replicate. For Fig. 2a and Supplementary Fig. 1e, images are representative examples from 2 independent biological replicates with *n* ≥ 50 condensates per replicate. For Figs. 2b–d, 7b, c, 8b, d images are representative examples from 3 independent biological replicates with at least *n* ≥ 10 U2OS cells per replicate. For Fig. 3b, images are representative examples from 3 independent biological replicates with *n* ≥ 100 *Caulobacter* cells per replicate and *n* ≥ 10 U2OS cells per replicate.

### Reporting summary

Further information on research design is available in the Nature Research Reporting Summary linked to this article.

## Supplementary information

Supplementary Information

Description of Additional Supplementary Files

Reporting Summary

Supplementary Data 1

Supplementary Data 2

Supplementary Video 1

## Data Availability

Processed tomograms generated as part of this study are available in the Electron Microscopy Data Resource under accession codes EMD-23622, EMD-23623, and EMD-23624. The unprocessed tilt series are available in the Electron Microscopy Public Image Archive (EMPIAR) under accession codes EMPIAR-10693, EMPIAR-10688 and EMPIAR-10689. Raw data used for bioinformatics analysis, ChpT localization images, and data generated from Monte Carlo simulations has been deposited at https://zenodo.org/record/7044613#.YyfqROzMLvU^90^. All other data supporting the findings of this study are included in the main text and the supplementary information. Strains and plasmids, supporting the findings of this study are available from the corresponding author(s). Source data are provided with this paper.

## Source data

Source Data

## Code availability

Simulations and simulation analysis were performed with open-source tools: campari V2 and ABSINTH: abs3.2_opls.prm (http://campari.sourceforge.net), SOURSOP 0.1.9 (https://soursop.readthedocs.io/), and MDTraj 1.9.5 (http://mdtraj.org/).
