# Supplementary material for: The material properties of a bacterial-derived biomolecular condensate tune biological function in natural and synthetic systems
Source: Nat Commun. 2022 Sep 26;13:5643. doi: 10.1038/s41467-022-33221-z (PMC9512792; doi:10.1038/s41467-022-33221-z)
Supplement: Supplementary file 1 — Supplementary Information [file 41467_2022_33221_MOESM1_ESM.pdf]

SUPPLEMENTARY INFORMATION

The material properties of a bacterial-derived biomolecular condensate tune biological function in natural and synthetic systems

Keren Lasker[1,2,*,#], Steven Boeynaems[3,*], Vinson Lam[4], Daniel Scholl[2], Emma Stainton[1], Adam Briner[5], Maarten Jacquemyn[6], Dirk Daelemans[6], Ashok Deniz[2], Elizabeth Villa[4], Alex S. Holehouse[7,8], Aaron D. Gitler[3,#], Lucy Shapiro[1,#]

1. Department of Developmental Biology, Stanford University School of Medicine, Stanford, CA 94305, USA
2. Department of Integrative Structural and Computational Biology, The Scripps Research Institute, La Jolla, CA 92037, USA
3. Department of Genetics, Stanford University School of Medicine, Stanford, CA 94305, USA
4. Division of Biological Sciences, University of California San Diego, La Jolla, CA 92093, USA
5. Clem Jones Centre for Ageing Dementia Research (CJCADR), Queensland Brain Institute (QBI), The University of Queensland, Brisbane, QLD 4072, Australia
6. KU Leuven Department of Microbiology, Immunology, and Transplantation, Laboratory of Virology and Chemotherapy, Rega Institute, KU Leuven, 3000 Leuven, Belgium
7. Department of Biochemistry and Molecular Biophysics, Washington University in St. Louis, St. Louis, MO 63110, USA.
8. Center for Science and Engineering of Living Systems (CSELS), Washington University in St. Louis, St. Louis, MO 63130, USA

* Equal contribution

\# Co-corresponding authors

Supplementary Notes

Supplementary note 1: PopZ concentration at the poles of the *Caulobacter* cell

PopZ is present at an approximately constant copy number over the *Caulobacter* cell cycle, and we estimate there are, on average, 3,500 ± 200 molecules of PopZ per cell[1,2]. Assuming the molecules are split between the two poles, each pole holds $1.66 * 10^{-21}$ moles of PopZ. The extent of the PopZ microdomain is 250 nm along the cell axis and 300 nm perpendicular to the cell axis[3], leading to an approximated volume of $3.92 \times 10^{-17}\ L$. Thus, the concentration of PopZ at the pole is approximately 50 μM.

Supplementary Figures

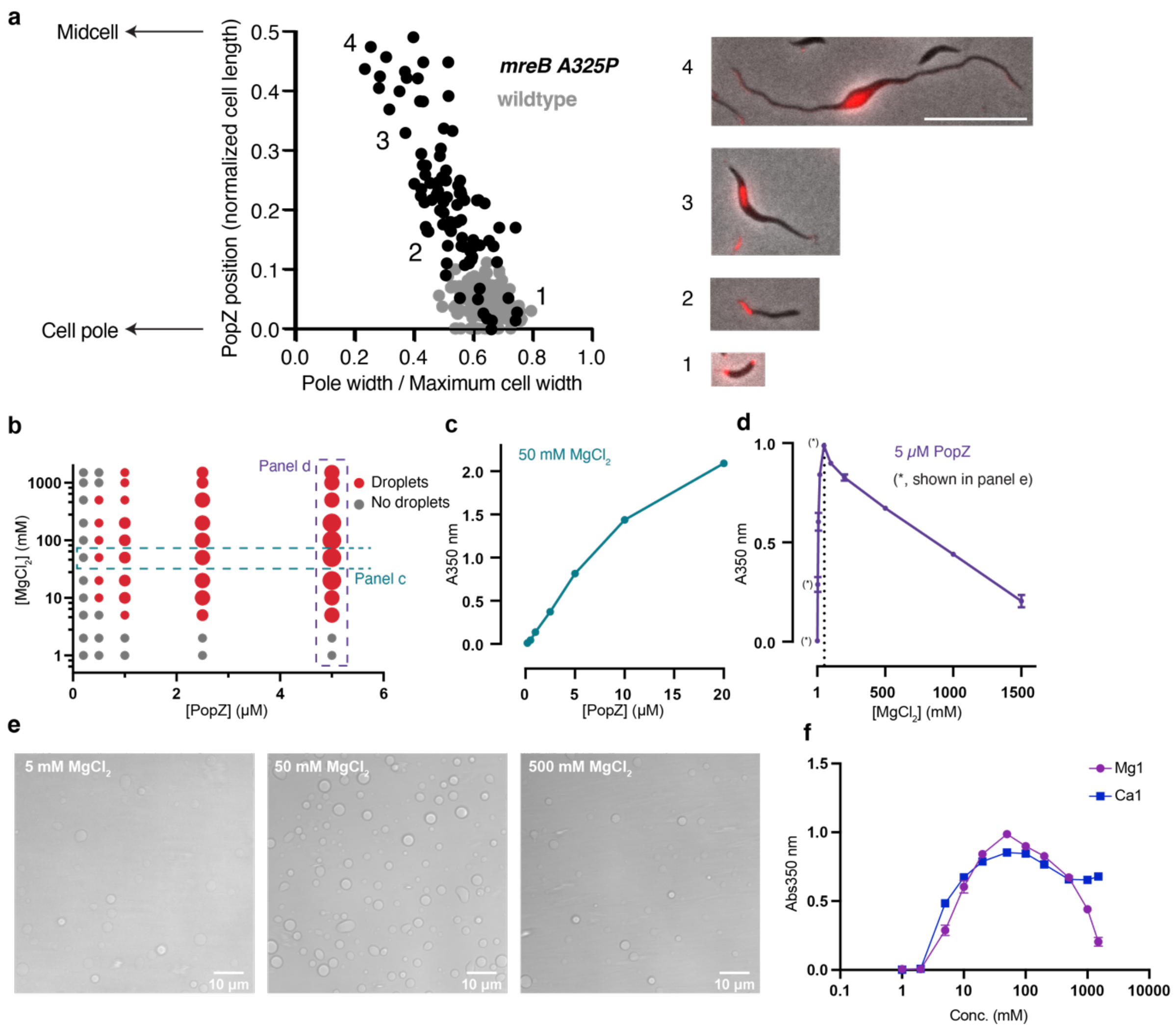

Supplementary figure 1. PopZ localization as a function of cell width and PopZ condensation in vitro.

a. Localization of the PopZ microdomain as a function of the cell width. (left) Scatter plot showing the PopZ microdomain's localization as a function of the ratio between the pole width and the maximum cell width (width ratio). Each dot corresponds to a single cell. In wildtype cells, the width ratio is maintained with a mean of 0.64 and SEM of 0.004 (gray violin plot), while in cells expressing *mreB* A325P, the width ratio varies with a mean of 0.5 and SEM of 0.01. In wildtype cells, there is a slight anti-correlation (cross-correlation score of -0.16) between PopZ localization and the width ratio (gray dots). In cells expressing *mreB* A325P, PopZ appears away from the pole

as the width ratio decreases, resulting from the thinning of the polar region and expansion of the cell body. This relationship is quantified by a strong anti-correlation (cross-correlation score of -0.80) between PopZ localization and the width ratio. Data from 80 cells. (right) Four snapshots of cells. Scalebar 10 µm. Source data underlying graphs are provided in Source Data. **b.** Phase diagram of PopZ condensation as a function of PopZ and $MgCl_2$ concentration. Turbidity was measured via absorbance at 350 nm. A_{350nm} values below 0.02 were considered to indicate the absence of droplets (gray circles), and higher values indicated the presence of droplets (red circles; the size of circles relates to the intensity of light scattering). Source data underlying graphs are provided in Source Data. **c-d.** Dependence of turbidity on PopZ concentration (c) and $MgCl_2$ concentration (d). Error bars represent the standard deviation of n=2 independent experiments. The asterisks in panel (d) refer to the conditions shown in panel (e). Source data underlying graphs are provided in Source Data. **e.** Differential interference contrast microscopy images of 5 µM PopZ under different conditions of $MgCl_2$ concentrations. **f.** $CaCl_2$ shows a similar effect as $MgCl_2$. PopZ concentration was kept constant at 5 µM. Experiments shown in panels b-f were performed in 5 mM sodium phosphate at pH 6.0. Source data underlying graphs are provided in Source Data.

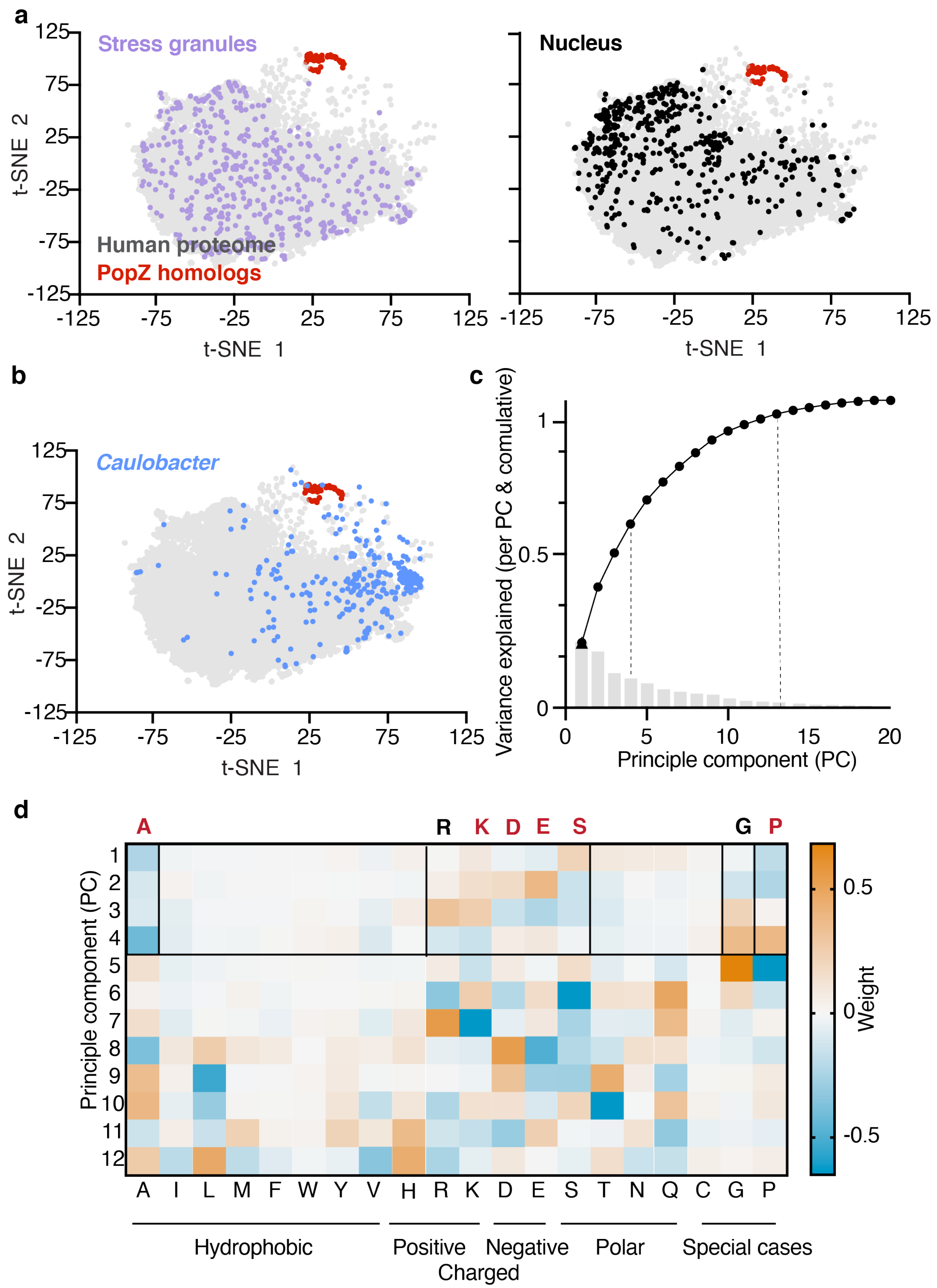
a
Stress granules
Human proteome
PopZ homologs
Nucleus
t-SNE 2
t-SNE 1
125
75
25
-25
-75
-125
b
Caulobacter
c
Variance explained (per PC & comulative)
Principle component (PC)
1
0.5
0
5
10
15
20
d
A
R
K
D
E
S
G
P
Principle component (PC)
Weight
0.5
0
-0.5
A I L M F W Y V H R K D E S T N Q C G P
Hydrophobic
Positive
Negative
Charged
Polar
Special cases

Supplementary figure 2. A comparison between IDRs in human cells and the PopZ IDR.

a. PopZ IDR sequence composition differs from IDRs found in human proteins. Shown are t-SNE mappings of IDR sequence composition. Each data point corresponds to the sequence composition of a single IDR. In gray are IDRs from the human proteome, and in red are IDRs from PopZ homologs within the *Caulobacterales* order. IDRs found in human stress granules proteins are shown in purple (left), and IDRs found in the nucleolus are shown in black (right). **b.** PopZ sequence composition is separate from most IDRs found in *Caulobacter crescentus*. The composition of IDRs found in *Caulobacter* proteins (blue) intersects with the composition of IDRs found in the human proteome (gray), unlike PopZ (red). **c-d.** Eight amino acids explain 60% of the variance in sequence composition across human and *Caulobacter* IDRs**.** c. Results of a principal component dimensionality reduction using the twenty amino acids as features. The first three (four) PCs explain 50% (60%) of the variance in the data, and 12 PCs are required to explain 95% of the variance in the dataset. **d.** Graphical representation of the 12 PCs used for the t-SNE analysis in panels a and b. The first four PCs use a linear combination of eight amino acids (black highlight), including polar residues (Asp, Glu, Lys, Arg), Ala, Pro, Gly, and Ser, which are enriched in IDRs of RNA binding proteins[4,5]. Amino acids that are present in PopZ IDR are highlighted in red. PopZ IDR is enriched in Asp, Glu, Ala, and Pro and lacks Arg, Lys, and Gly.

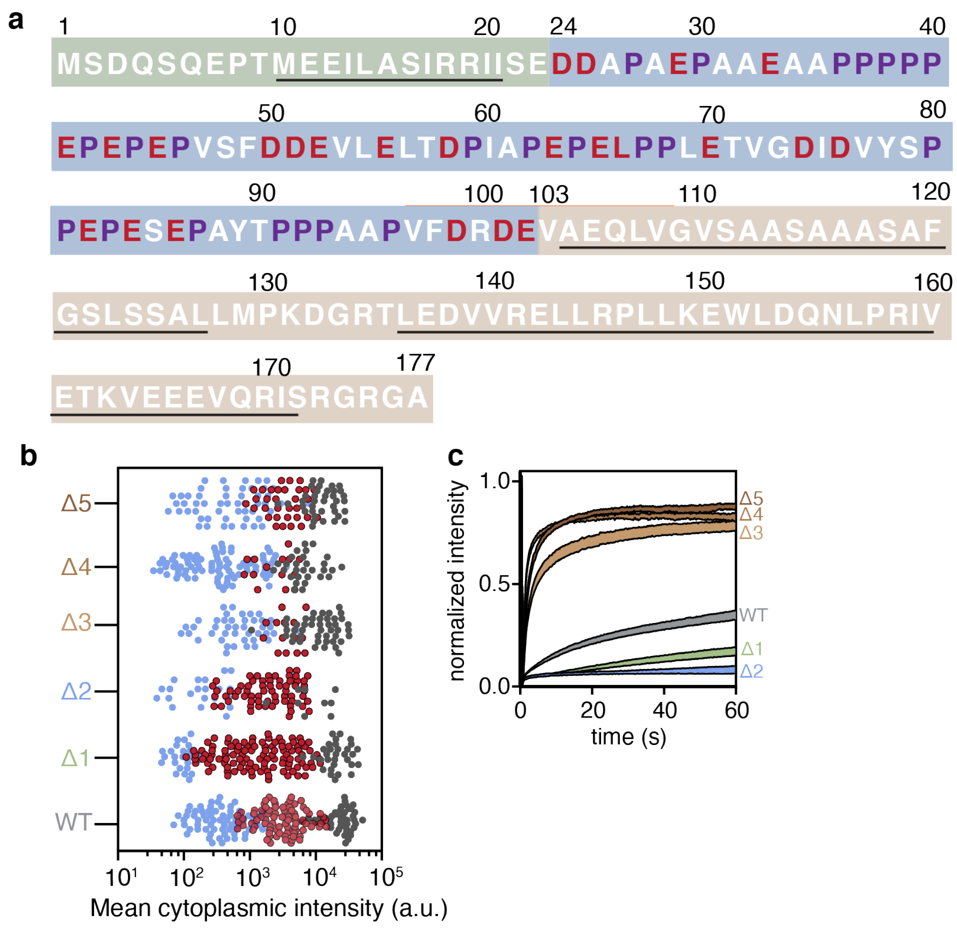

Supplementary figure 3. Characterizing the condensation of PopZ truncations

a. PopZ primary sequence. The N-terminal, IDR, and C-terminal regions are indicated above using a green, blue, and brown background. The IDR's prolines are colored in purple and negatively charged residues in red. Black rectangles indicate the boundaries of α-helices as predicted using the MPI Bioinformatics Toolkit[6] **b.** Phase diagram of PopZ truncations. Phase diagrams of EGFP fused to wildtype PopZ or one of five PopZ truncations, as indicated in Figure 2. Each dot represents data from a single cell, positioned on the x-axis as a function of the cell mean cytoplasmic intensity. The color of the dot indicates its phase, a dilute phase (blue), two-phase (red), or dense phase (gray). **c.** FRAP of PopZ truncations. Fluorescence recovery after photobleaching (FRAP) curves for wildtype PopZ and the five truncations. This is a different representation of the data shown in Fig. 3c. Deleting either region 1 or the IDR leads to reduced recovery. In contrast, deletion of any of the three helical c-terminal regions leads to increased mobility. Source data underlying graphs are provided in Source Data.

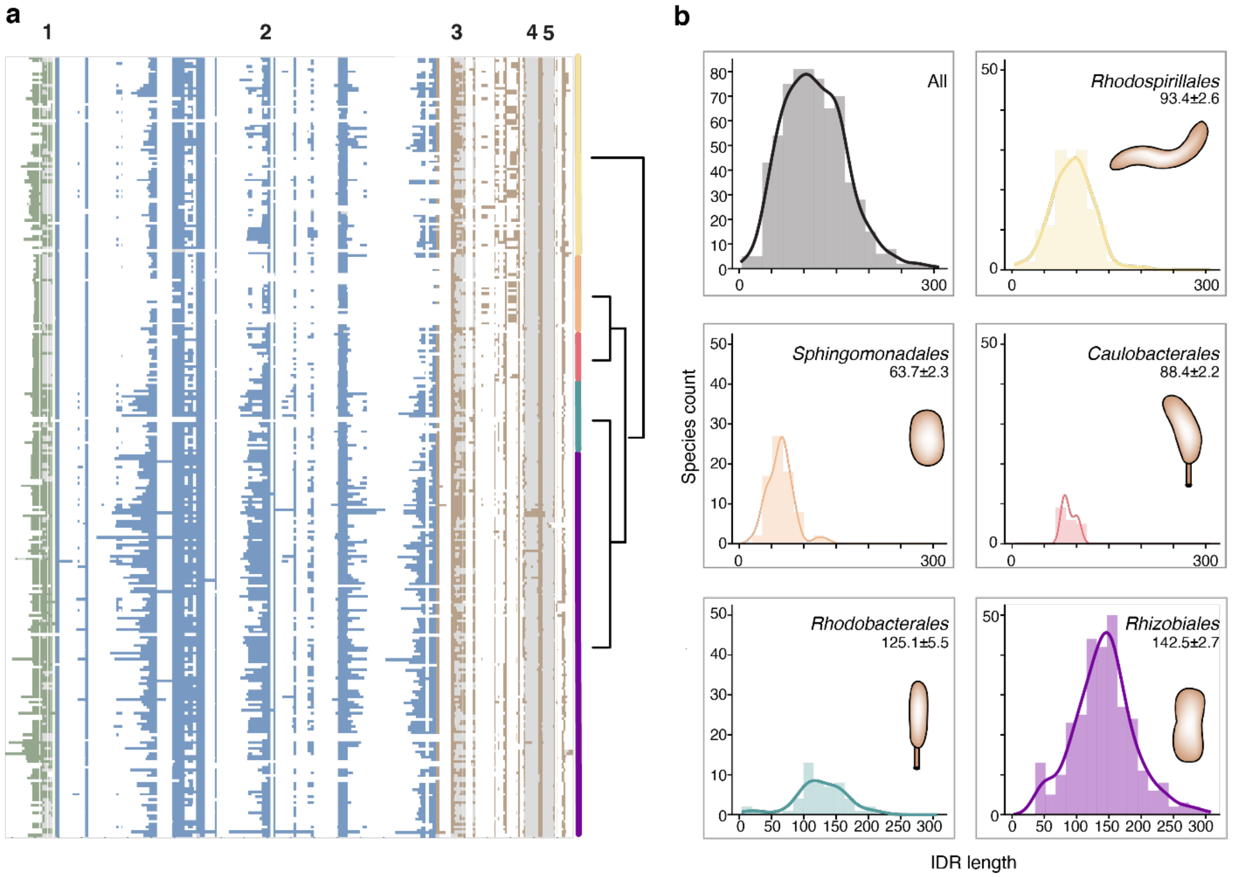

Supplementary figure 4. PopZ sequence across alpha-proteobacteria.

a. Conservation of the PopZ protein regions within α-proteobacteria. Graphical representation of an alignment of 655 PopZ homologs across α-proteobacteria. Each row corresponds to a PopZ homolog, and each column to an alignment position. All PopZ homologs encode a short helical N-terminal region (green), an IDR (blue), and a helical C-terminal helical region (brown). The C-terminal region is divided into two sub-modules: a region that includes helix 2, which varies in length and helicity, and a region that includes helices 3 and 4, which is highly conserved. White regions indicate alignment gaps, and gray regions indicate predicted helices 1 to 4. Phylogeny tree of the corresponding species is shown, highlighting five major orders within α-proteobacteria: *Rhodospirillales* (yellow), *Sphingomonadales* (orange), *Caulobacterales* (red), *Rhodobacterales* (green), and *Rhizobiales* (purple). **b.** A wide distribution of linker length across α-proteobacteria. Shown is the length distribution of the PopZ IDR across all 655 representatives α-proteobacteria per order. Mean and SEM is reported for each.

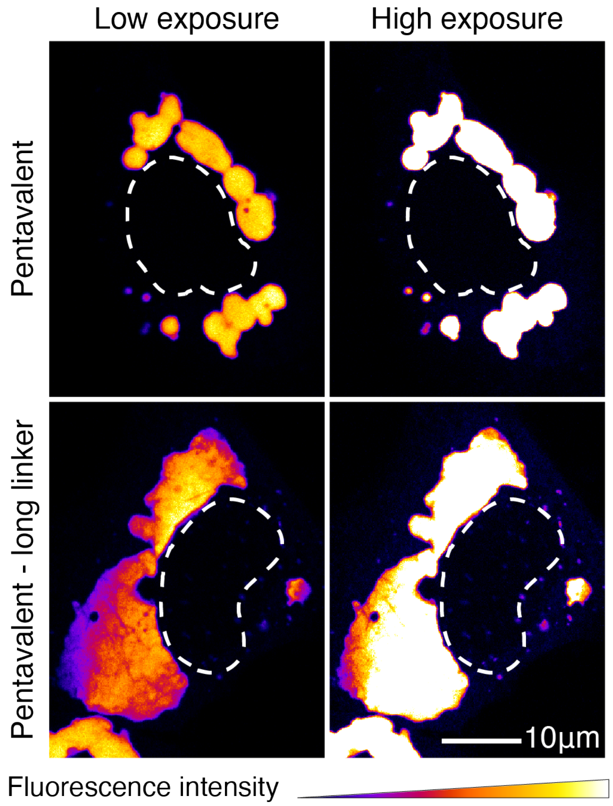

Supplementary figure 5. Pentavalent PopZ forms dense irregular condensates and Pentavalent PopZ with a long linker forms a large condensate.

EGFP fused to pentavalent PopZ (top) and pentavalent PopZ with a long linker (bottom) expressed in human U2OS cells. Data are shown with low exposure (left) and high exposure (right). A color gradient indicates EGFP fluorescence intensity from blue (low) to white (high). The nucleus boundary is shown as a white dotted line. Pentavalent PopZ forms small dense and irregular condensates. Pentavalent PopZ with a long linker forms one large condensate that takes over most of the cytosol. Scale bar, 10 µm.

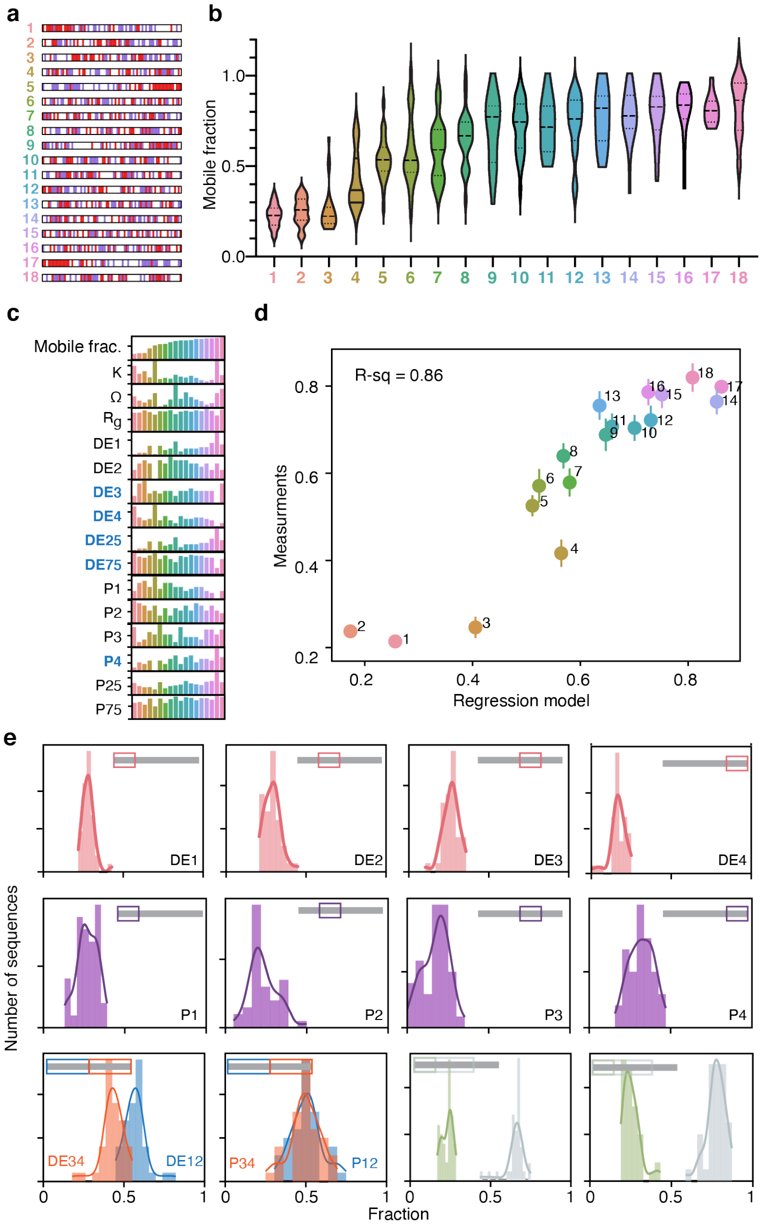

Supplementary figure 6. Charge distribution contributes to PopZ liquidity.

a. A graphical representation of 18 scrambles of the wildtype PopZ IDR. Red indicates an acidic amino acid, and purple indicates a proline. The scrambles are sorted by their mobile fraction. **b.** A violin plot of FRAP measurements of PopZ condensates in U2OS cells. The FRAP data are shown as mobile fractions, which vary from 30% (for L1), 63% (for the wildtype IDR L8), to 80% (for L18). *n* equals 25 granules per condition. **c.** 16 Bar plots, each shows the values of a feature across the 18 scrambles. The bars are color-coded by the scramble numbers (a). Features include (i) FRAP mobile fraction, (ii) $0 \leq K \leq 1$ parametrizing the degree of mixing (0) *vs.* segregation (1) of

oppositely charged residues[7], (iii) $0 \leq \Omega \leq 1$ parametrizing the degree of mixing (0) *vs.* segregation (1) of charged and proline residues[8], (iv) R_g radius of gyration as calculated by an all-atom-simulation (Methods), (v) 0 ≤DE1-4≤ 1, the fraction of acidic residues in each quarter of the sequence, (vi) 0 ≤DE25, DE75≤ 1, N-terminal fraction that includes 25% and 75% of the IDR acidic residues, (vii) 0 ≤P1-4≤ 1, the fraction of proline residues in each quarter of the sequence, (viii) 0 ≤P25, P75≤ 1, N-terminal fraction that includes 25% and 75% of the IDR prolines. **d.** A regression model using 5 of 25 features derived from the IDR sequence (Methods). The model presented has an R-square of 0.86 and is a linear combination of differential N- versus C- acidity, as well as the number of prolines present in the C-terminal quarter of the IDR (DE3, DE4, DE25, DE75, P4). Shown are the predicted mobile fraction for the 18 PopZ mutants as calculated by the regression model (x-axis) compared to the measured mobile fraction (y-axis, same data as (a) presented as mean +/- SEM, *n* equals 25 granules per PopZ mutant). **e.** The features used in the regression model are conserved across *Caulobacterales*. The histograms are calculated across 99 PopZ homologs within the *Caulobacterales* order.

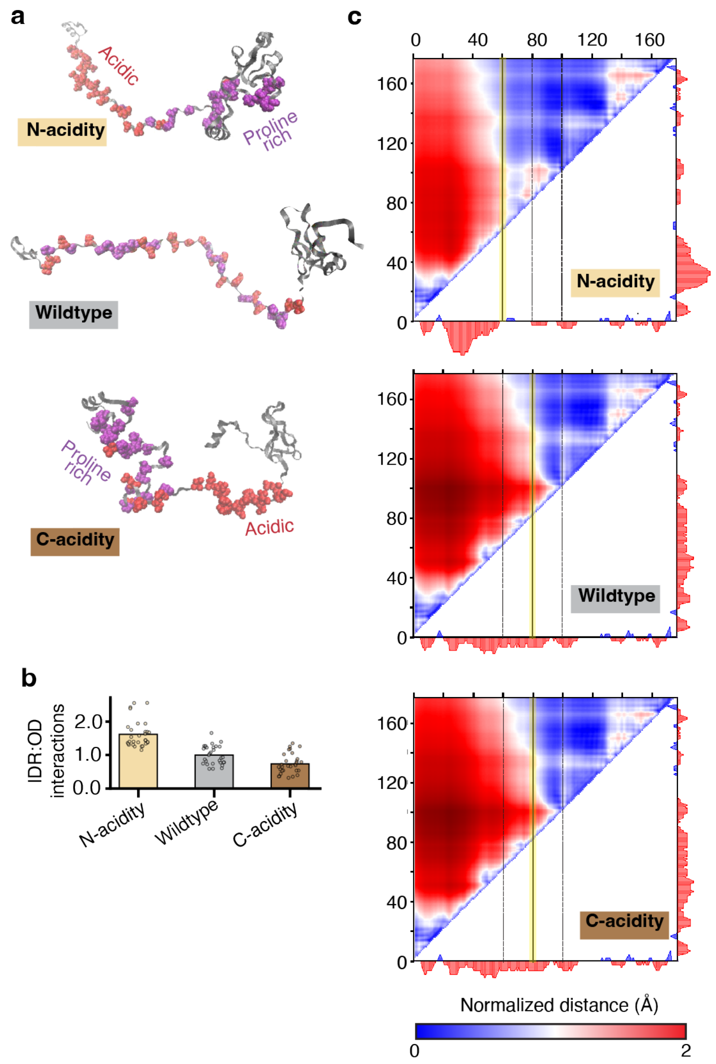

Supplementary figure 7. All-atom simulations suggest that competing IDR-OD and OD-OD interactions can regulate the dynamics of PopZ condensates

a. Snapshots of all-atom simulations. PopZ condensation is driven by OD-OD interactions (Fig. 3a). We wondered if the relative position of the acidic residues in the linker could modulate OD-OD interactions. We simulated the dynamics of wildtype PopZ, C-acidity PopZ, which has its acidic residues segregated to the C-terminal end of the IDR, and N-acidity PopZ, which is the reverse sequence with all of the acidic residues segregated to the N-terminal end of the linker. **b-c.** All-atom simulations suggest differences in IDR/OD interactions as a function of the distribution of charged residues. (b) The fraction of conformations in which the IDR interacts with the OD for PopZ, C-acidity PopZ, and N-acidity PopZ. *n* equals 30 independent simulations for each of these PopZ variants. The data is presented as a bar graph, overlayed with the corresponding data points,

and normalized to wildtype simulation. The N-acidity IDR interacts more with its adjacent OD compared to the wildtype IDR, while the C-acidity IDR tends to interact less with its adjacent OD. These findings suggest that competing IDR-OD and OD-OD interactions can regulate the dynamics of PopZ condensates. (c) Per residue distance matrices show the average distance between pairs of residues in wildtype PopZ, N-acidity, and C-acidity.

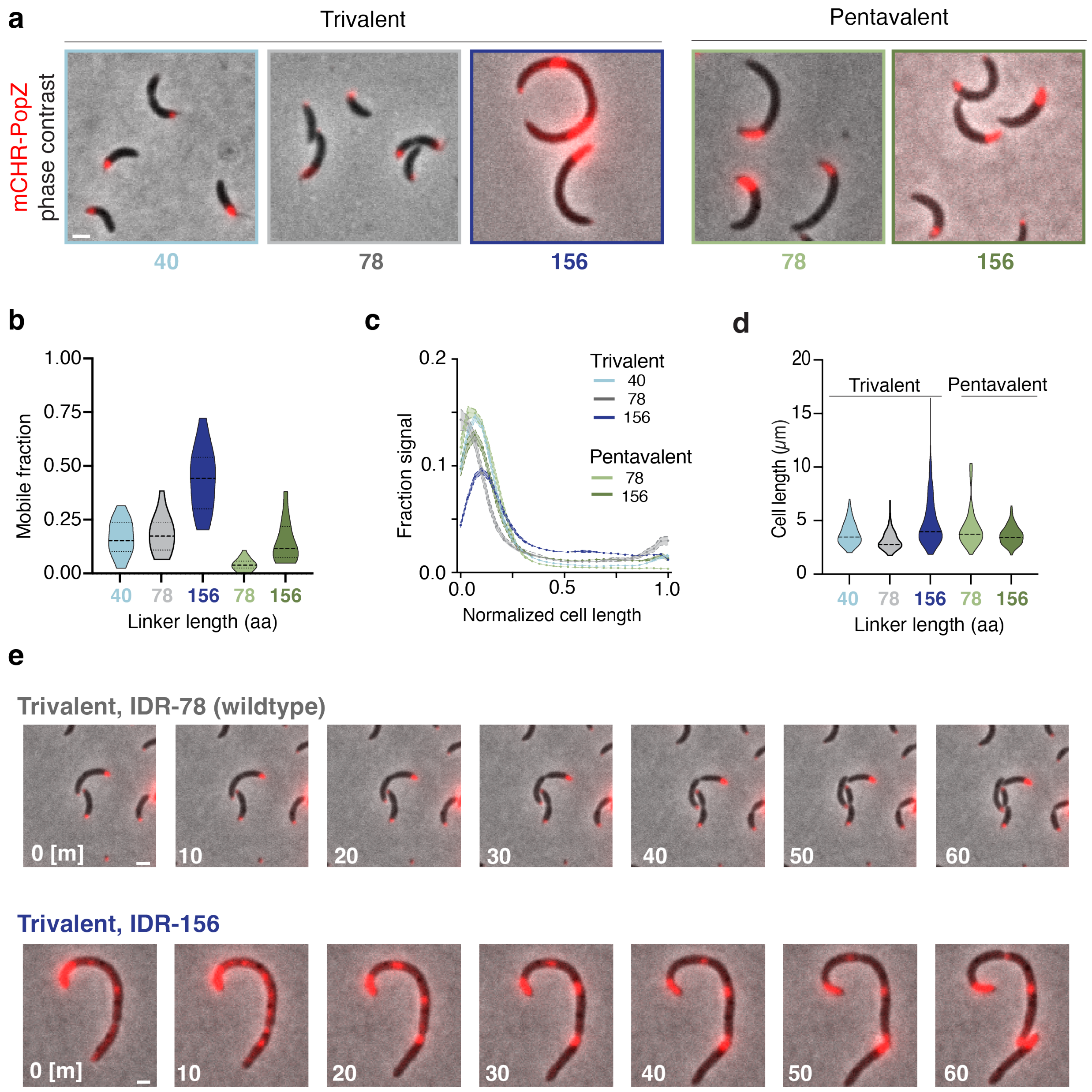

Supplementary figure 8. IDR length and OD valency affect PopZ localization.

a. Linker length and its effect on condensate localization in *Caulobacter*. ΔpopZ *Caulobacter* cells expressing mCherry fused to PopZ with an IDR of different lengths and either a trivalent or a pentavalent c-terminal region. mCherry-PopZ with IDR-40 or the wildtype IDR-78 maintains its localization at the poles of the cell, while mCherry-PopZ with IDR-156 demonstrates condensates throughout the cytoplasm. The mutants of PopZ with pentavalent c-term both show polar localization. Scale bar, 1 μm. **b.** The balance between condensation promoting and obstructing tunes material properties. A violin plot of the distribution of FRAP measurements for the different mutants in *Caulobacter*. FRAP, shown as mobile fractions, for PopZ with its wildtype oligomerization domain (trivalent) and a linker of three different lengths (shades of blue and gray), as well as PopZ with an extended oligomerization domain (pentavalent) with IDR-78 (light green) and IDR-156 (dark green). **c.** Fluorescence intensity profiles along normalized cell length for ΔpopZ *Caulobacter* cell overexpressing different PopZ mutants (n = 51, 250, 250, 164, and 142 for trivalent OD with IDR-40, IDR-78, IDR-156, and pentavalent OD with IDR-78 and IDR-156). Cells expressing wildtype PopZ show microdomains at both poles. In cells expressing IDR-78 with pentavalent OD, a second polar PopZ microdomain is lost and recovered in IDR-156 with pentavalent OD. Source data underlying graphs are provided in Source Data. **d.** Cell length for the different mutants. A violin plot of the distribution of cell lengths for the different mutants. At least 30 cells were measured for each condition. **e.** Time-lapse showing 10 minutes snapshots of ΔpopZ *Caulobacter* cells expressing wildtype PopZ and IDR-156. Scale bar, 1 μm.

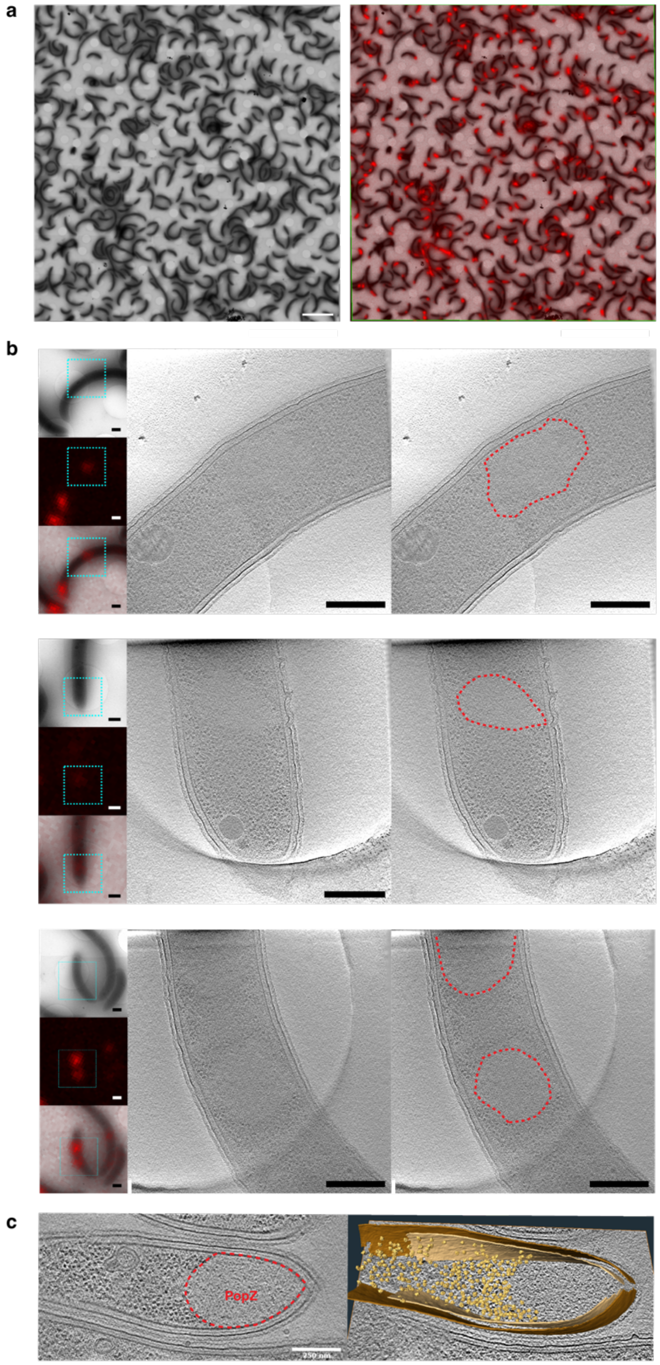

Supplementary figure 9. PopZ mutants with IDR-156 maintain ribosome exclusion

a-b. Correlative fluorescence cryo-electron tomography imaging of cells expressing mCherry-PopZ with IDR-156. a. A field of view of cells on a cryo-grid imaged using an electron microscope (left) and a fluorescence microscope in cryo-conditions (right). PopZ is shown in red. Scale bar, 10 μm. b. In each row shown are (left, top) a cell in low-magnification with a blue square indicating

the region for high-magnification tomography imaging, (left, middle) the same low-magnification imaging area in cryo-fluorescence, and (left, bottom) an overlay of the two channels. (middle) a slice of the reconstructed tomogram showing a ribosome-free region. (right) the same slice as in (middle) with the PopZ region annotated, as resolved from the fluorescence channel. Scale bar, 0.5 μm. **c.** (left) Slice through a tomogram of a cryo-focused ion beam-thinned ΔpopZ *Caulobacter* cell overexpressing mCherry-PopZ with IDR-156 and pentavalent OD. (right) Segmentation of the tomogram in (left) showing annotated outer membrane (dark brown), inner membrane (light brown), and ribosomes (gold). Scale bar, 0.25 μm.

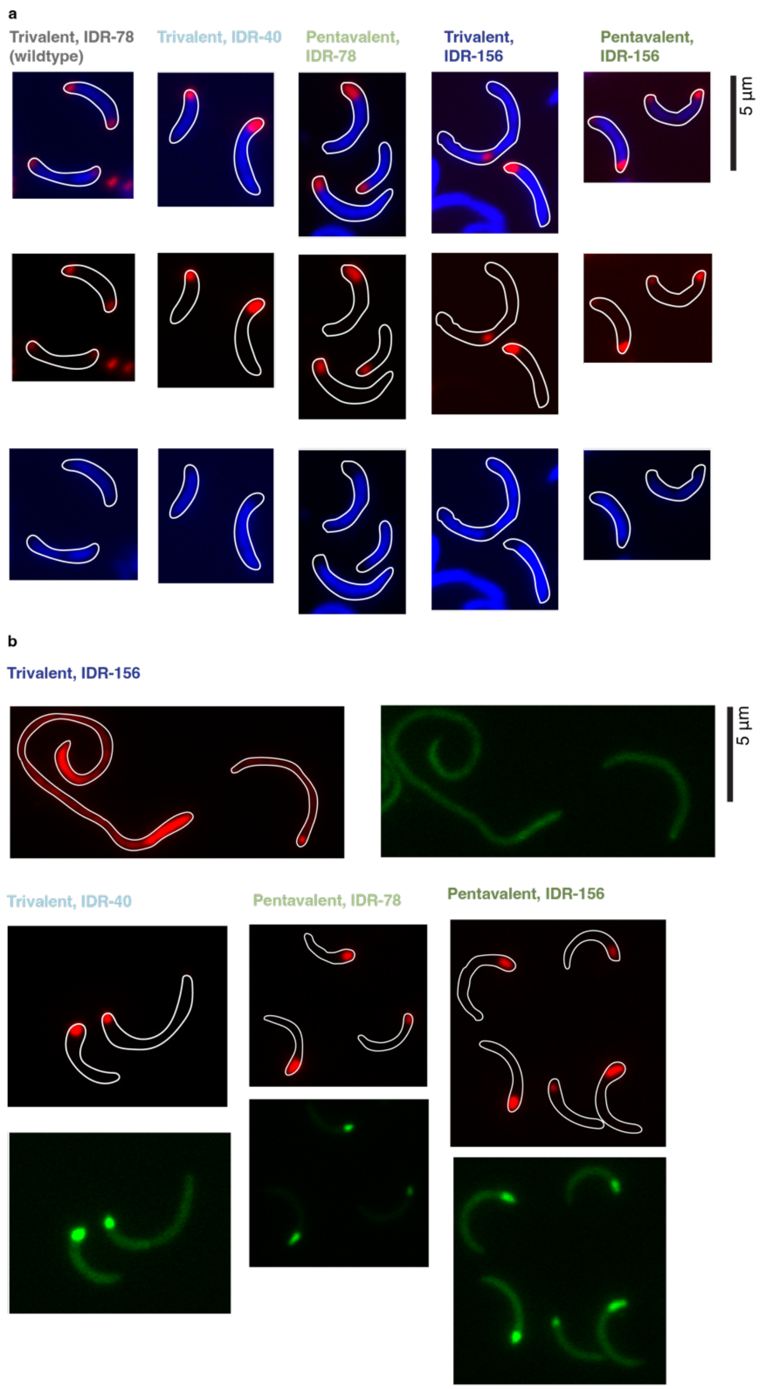

Supplementary figure 10. While DNA exclusion is maintained across different IDR lengths and OD valencies, client recruitment is affected.

a. DNA exclusion is maintained. ΔpopZ *Caulobacter* cells expressing mCherry fused to PopZ with an IDR of different lengths and a trivalent or a pentavalent c-terminal region. The cells were stained with DAPI, washed, and imaged immediately thereafter. DNA exclusion (blue) was

observed for all PopZ mutants (red). In liquid droplets that left the pole (IDR-156), we observed DNA penetrating the polar regions while creating DNA voided regions in the cytosolic regions occupied by PopZ. Scale bar, 5 µm. **b.** Client recruitment is impaired. ΔpopZ *Caulobacter* cells in which the native ChpT gene was replaced with eYFP-ChpT expressing mCherry fused to PopZ mutants as in (a). Quantification of the observed partition coefficient is shown in Figure 3e. Scale bar, 5 µm.

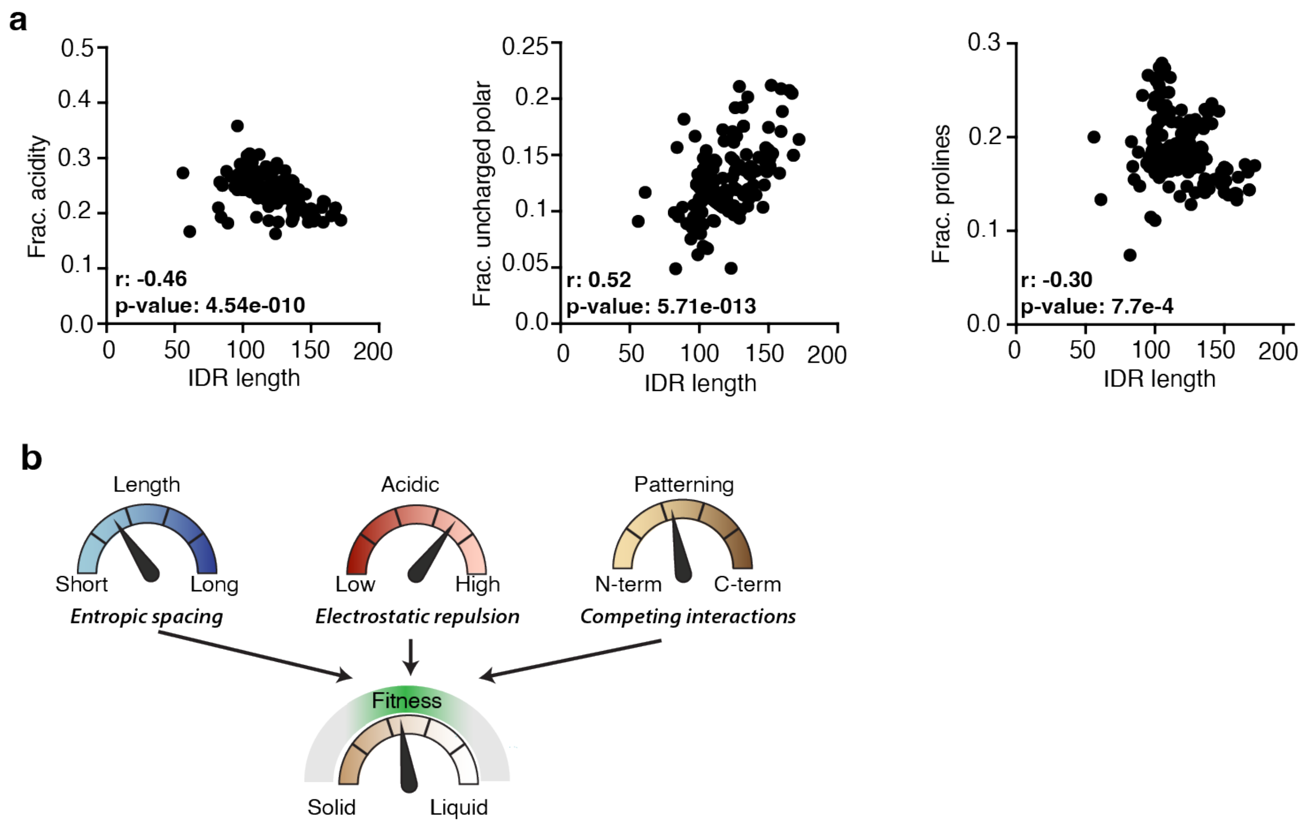

Supplementary figure 11. Evolutionary compensation of IDR properties.

a. PopZ homologs with longer IDR tend to have a lower fraction of acidic residues. Scatter plot showing the relation between IDR length and acidity across *Caulobacterales.* (left) fraction acidity as a function of IDR length shows a reduced fraction of acidic residues as the length of the IDR increases. (middle) it also shows an increase in polar non-charged residues (N, C, Q, S, and T). (right) this trend does not hold for fraction prolines. For each, Pearson correlation (r) and p-value

are indicated. Source data underlying graphs are provided in Source Data. **b.** Evolutionarily conserved knobs that tune PopZ material properties are linked to fitness. Chart illustrating the three identified IDR sequence features (IDR length in blue, IDR composition in red and acidity partitioning in yellow) and their modulation of PopZ material properties (solid to liquid, brown). These material properties, in turn, are linked to cell fitness (green).

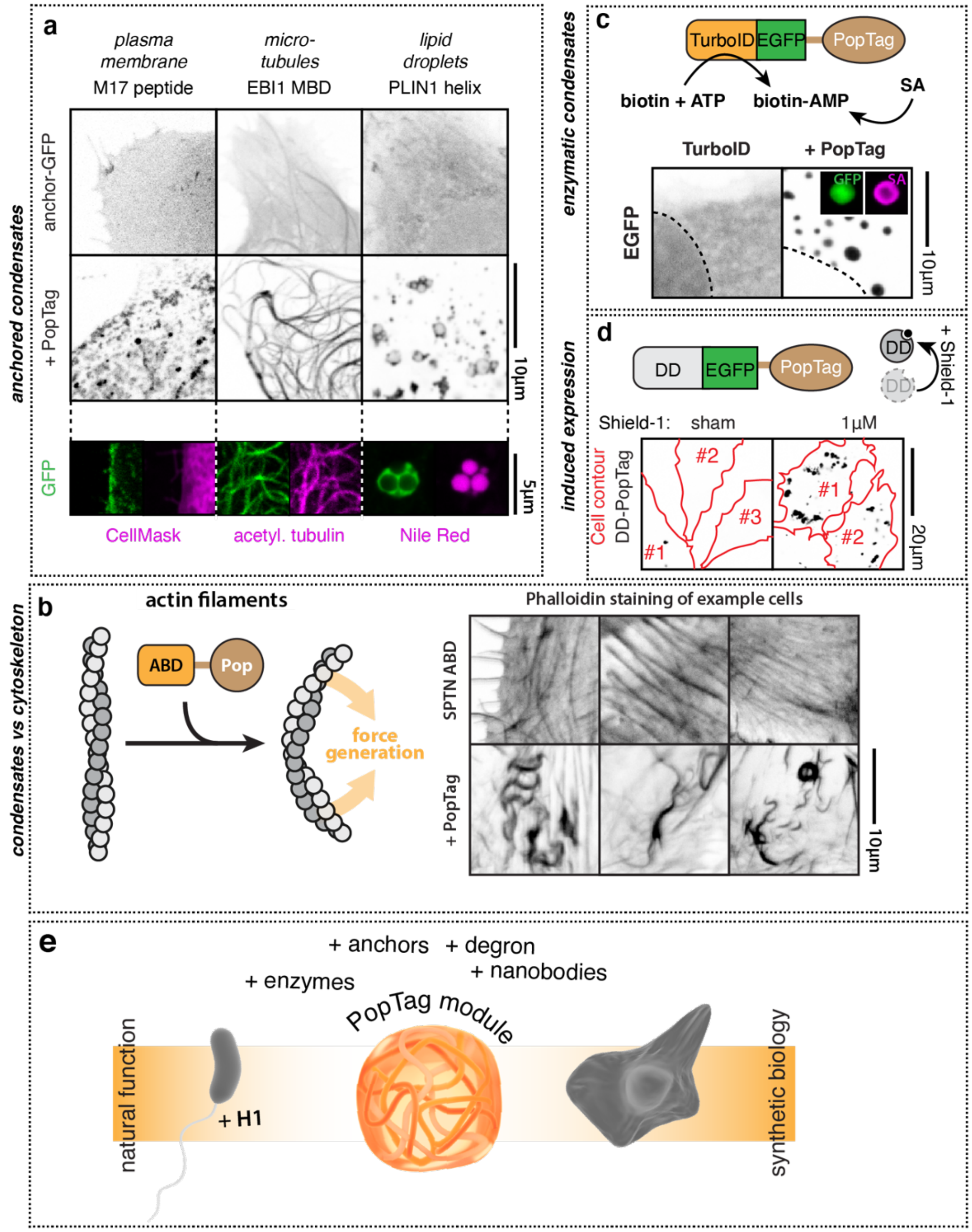

Supplementary figure 12. PopTag condensates have tunable functionality.

a. Subcellular anchors control PopTag condensate localization. Each anchor peptide is fused to EGFP in the presence or absence of the added PopTag. The M17 peptide is derived from the HIV Gag protein, the microtubule-binding domain (MBD) is from EBI1, and the amphipathic helix is from PLIN1. CellMask labels the plasma membrane (purple), acetylated tubulin labels microtubules (purple), and Nile Red labels lipid droplets (purple). Peptide-EGFP fused to PopTag is shown in green. **b.** Condensation of the PopTag on actin filaments by fusion to β-spectrin actin-binding domain (SPTN ABD) drives coalescence, buckling, and bending of actin filaments (stained with phalloidin), similar to the effect of molecular motors on actin filaments[9]. Our results indicate that cytoplasmic condensates can exert force on the cytoskeleton, akin to nuclear bodies interacting with the genome[10] and TIS-granules embedded between endoplasmic reticulum tubules[11]. **c.** Turbo-ID[12] fused to PopTag generates enzymatic microreactors and retains enzymatic biotinylation activity as indicated by streptavidin (SA) staining. **d.** Fusing PopTag to the drug-stabilized degron[13] (DD) allows for the pharmacological control of PopTag expression. The addition of Shield-1 stabilizes the degron and prevents degradation of DD-PopTag condensates. **e.** Scheme highlighting how different actor domains drive PopZ/PopTag function in nature or synthetic biology. The examples shown in Fig. 7d-e and Extended Data Fig. 11a-d demonstrate that the modular architecture of PopZ as discovered in *Caulobacter* is maintained when expressed in human cells and allows fusion of the PopTag to different actor domains and different PopZ IDRs to build an array of functionalized condensates.

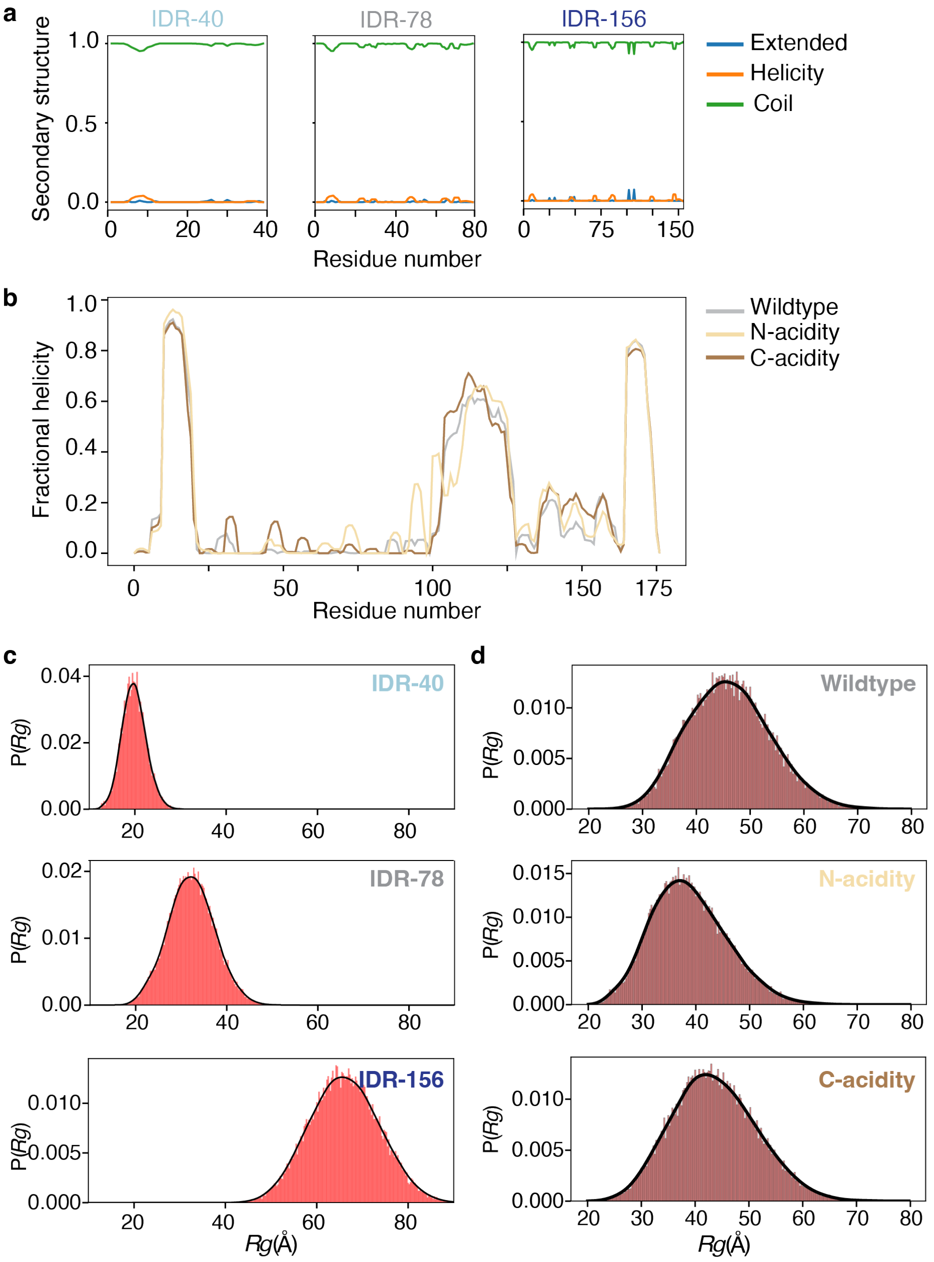

Supplementary figure 13. Simulations show a complete sampling

a. Secondary structure analysis for the IDR-40 (half), IDR-78 (full), and IDR-156 (double) PopZ linkers reveals no significant helicity or beta-sheeted (extended) structure. **b.** Comparative helicity analysis for WT PopZ and the N-acidity and C-acidity mutants. **c.** Distributions for the radii of gyration of IDR-40 (half), IDR-78 (full), and IDR-156 (double) PopZ linkers. The broad, smooth distributions match expectations for a flexible polymer, suggesting robust and effective conformational sampling. **d.** Distributions for the radius of gyration of WT PopZ and the two linker charge mutants.

Supplementary Tables

Supplementary table 1. Overview of simulation input settings

System	Droplet radius (Å)	Total number of replicas	Steps per simulation	Equilibration steps	Conformations per simulation	Total ensemble size
WT linker	122	30	60 000 000	6 000 000	1200	36 000
Half linker	81	5	27 000 000	2 000 000	1250	6 250
Double linker	184	30	120 000 000	6 000 000	1200	34 500
Full protein	200	30	150 000 000	10 000 000	1500	45 000
Full (N terminal charge block)	200	30	150 000 000	10 000 000	1500	45, 000
Full (C terminal charge block)	200	30	150 000 000	10 000 000	1500	45 000
Sequence length titrations	Variable	20	66 000 000	4 000 000	1320	39, 600

Supplementary table 2. Summary of simulation details for all-atom simulations.

System	N_{res}	Mean R_g (Å)	Mean R_e (Å)	ν^{app}
Wildtype linker	78	32.4	90.6	0.72
Half linker	39	19.9	49.3	0.61
Double linker	154	66.2	182.8	0.78
Full protein	177	46.3	107.7	0.64
Full (N terminal charge block)	177	38.9	92.5	0.58
Full (C terminal charge block)	177	43.8	96.7	0.65

The apparent scaling exponent (ν^{app}) was calculated by fitting a linear model to the intra-residue distances, as described previously[14]. The fitting of ν^{app} becomes systematically less useful and appropriate as a chain deviates from homopolymer behavior (as has been extensively discussed previously) and as such specific values are relatively uninformative for the full-protein constructs[15].

Supplementary table 3. ***Caulobacter*** **plasmids**

**Plasmids**			
**Number**	**Description**	**Strain number**	**Source**
AP211	pBXMCS-2, mCherry-PopZ. High copy plasmid (kanr)	AP211	[16]
pKL539	pBXMCS-2, mCherry-PopZ with IDR-48	KL6254	This study
pKL540	pBXMCS-2, mCherry-PopZ with IDR-156	KL6252	This study
pKL577	pBXMCS-2, mCherry-PopZ with IDR-78 and pentavalent OD	KL6322	This study
pKL581	pBXMCS-2, mCherry-PopZ with IDR-156 and pentavalent OD	KL6326	This study
pKL699	mCherry-PopZ with L5	KL6607	This study
pKL700	mCherry-PopZ with L17	KL6531	This study
pKL702	pBXMCS-2, mCherry-PopZ with IDR 100% P-G	KL6529	This study
pKL704	pBXMCS-2, mCherry-PopZ with IDR 100% DE-N	KL6530	This study
pKL703	pBXMCS-2, mCherry-PopZ with IDR 50% DE-N	KL6554	This study

Supplementary table 4. ***Caulobacter*** **strains**

**Strains**		
**Number**	**Description**	**Source**
KL5820	Δ*popZ*	LS5130[17]
KL5943	*mreB*::A325P*mreB*	JAT702[18]
KL6212	AP211 in *mreB*::A325P*mreB*	This study
KL6256	PopZ with IDR-40 in Δ*popZ*.	pKL539 electroporated into KL5820
KL6261	PopZ with IDR-156 in Δ*popZ*.	pKL540 electroporated into KL5820
KL6341	mCherry-PopZ with IDR-78 and pentavalent OD in Δ*popZ*.	pKL577 electroporated into KL5820
KL6349	mCherry-PopZ with IDR-156 and pentavalent OD in Δ*popZ*	pKL581 electroporated into KL5820
KL6561	mCherry-PopZ with L5 in Δ*popZ*	pKL699 electroporated into KL5820
KL6545	mCherry-PopZ with L17 in Δ*popZ*	pKL700 electroporated into KL5820
KL6541	mCherry-PopZ with all prolines replaced with glycines in Δ*popZ*	pKL702 electroporated into KL5820
KL6542	mCherry-PopZ with all acidic residues replaced with asparagines in Δ*popZ*	pKL704 electroporated into KL5820
KL6562	mCherry-PopZ with half of all acidic residues replaced with asparagines in Δ*popZ*	pKL703 electroporated into KL5820

Supplementary table 5. Cloning primers

Pair number	Forward	Reverse
1	GGGACGCGGCGCCTAAGAATTCCTGCAGCCCGGGGG	GGTTCTTGAGACTGATCGGACATGGTACCATGCATATTAATTAAGGCGCCTGC
2	CCTGTGTTTGACCGTGATGCCGAGCAGCTGGTCGGC	GGCTGGTGCATCATCCTCCGAGATGATGCGTCGAATGGAGG
3	CGTGGAGCTTAAGAATTCCTGCAGCCCGG	CGTCCACGAGAGATACGCTGCAC

Supplementary References

1 Aretakis, J. R., Gega, A. & Schrader, J. M. Absolute Measurements of mRNA Translation in Caulobacter crescentus Reveal Important Fitness Costs of Vitamin B12 Scavenging. *mSystems* **4** (2019).

2 Lasker, K. *et al.* Selective sequestration of signalling proteins in a membraneless organelle reinforces the spatial regulation of asymmetry in Caulobacter crescentus. *Nat Microbiol* **5**, 418-429 (2020).

3 Dahlberg, P. D. *et al.* Cryogenic single-molecule fluorescence annotations for electron tomography reveal in situ organization of key proteins in Caulobacter. *Proc Natl Acad Sci U S A* **117**, 13937-13944 (2020).

4 King, O. D., Gitler, A. D. & Shorter, J. The tip of the iceberg: RNA-binding proteins with prion-like domains in neurodegenerative disease. *Brain Res* **1462**, 61-80 (2012).

5 Kato, M. *et al.* Cell-free formation of RNA granules: low complexity sequence domains form dynamic fibers within hydrogels. *Cell* **149**, 753-767 (2012).

6 Stefani, J. *et al.* Disruption of the Microglial ADP Receptor P2Y13 Enhances Adult Hippocampal Neurogenesis. *Front Cell Neurosci* **12**, 134 (2018).

7 Das, R. K. & Pappu, R. V. Conformations of intrinsically disordered proteins are influenced by linear sequence distributions of oppositely charged residues. *Proc Natl Acad Sci U S A* **110**, 13392-13397 (2013).

8 Martin, E. W. *et al.* Sequence Determinants of the Conformational Properties of an Intrinsically Disordered Protein Prior to and upon Multisite Phosphorylation. *J Am Chem Soc* **138**, 15323-15335 (2016).

9 Murrell, M. P. & Gardel, M. L. F-actin buckling coordinates contractility and severing in a biomimetic actomyosin cortex. *Proc Natl Acad Sci U S A* **109**, 20820-20825 (2012).

10 Shin, Y. *et al.* Liquid Nuclear Condensates Mechanically Sense and Restructure the Genome. *Cell* **175**, 1481-1491 e1413 (2018).

11 Ma, W. *et al.* Unstructured mRNAs form multivalent RNA-RNA interactions to generate TIS granule networks. *bioRxiv*, 2020.2002.2014.949503 (2020).

12 Branon, T. C. *et al.* Efficient proximity labeling in living cells and organisms with TurboID. *Nat Biotechnol* **36**, 880-887 (2018).

13 Banaszynski, L. A. *et al.* A rapid, reversible, and tunable method to regulate protein function in living cells using synthetic small molecules. *Cell* **126**, 995-1004 (2006).

14 Peran, I. *et al.* Unfolded states under folding conditions accommodate sequence-specific conformational preferences with random coil-like dimensions. *Proc Natl Acad Sci U S A* **116**, 12301-12310 (2019).

15 Stenzoski, N. E. *et al.* The Cold-Unfolded State Is Expanded but Contains Long- and Medium-Range Contacts and Is Poorly Described by Homopolymer Models. *Biochemistry* **59**, 3290-3299 (2020).

16 Bowman, G. R. *et al.* Oligomerization and higher-order assembly contribute to sub-cellular localization of a bacterial scaffold. *Mol Microbiol* **90**, 776-795 (2013).

17 Bowman, G. R. *et al.* A polymeric protein anchors the chromosomal origin/ParB complex at a bacterial cell pole. *Cell* **134**, 945-955 (2008).

18 Dye, N. A. *et al.* Mutations in the nucleotide binding pocket of MreB can alter cell curvature and polar morphology in Caulobacter. *Mol Microbiol* **81**, 368-394 (2011).
