# Supplementary material for: The material properties of a bacterial-derived biomolecular condensate tune biological function in natural and synthetic systems
Source: Nat Commun. 2022 Sep 26;13:5643. doi: 10.1038/s41467-022-33221-z (PMC9512792; doi:10.1038/s41467-022-33221-z)
Supplement: Supplementary file 2 — Description of Additional Supplementary Files [file 41467_2022_33221_MOESM2_ESM.pdf]

Title: Supplementary Data 1.
Description: Sequences of PopZ mutants. Rows 1-22 list sequences of PopZ proteins with mutated IDRs. Rows 23-24 list the sequences of PopZ proteins with pentavalent oligomerization domains (ODs). IDR regions are shown in red, and pentavalent OD regions are shown in blue.

Title: Supplementary Data 2.
Description: Constructs used in the human cell lines studies.

Title: Supplementary Video 1.
Description: PopZ goes through fusion events when expressed in U2OS cells. EGFP-PopZ condensates undergo fusion events within a human cell. Image dimensions are 34x34um. The total time is 30 seconds.
