# Supplementary material for: The material properties of a bacterial-derived biomolecular condensate tune biological function in natural and synthetic systems
Source: Nat Commun. 2022 Sep 26;13:5643. doi: 10.1038/s41467-022-33221-z (PMC9512792; doi:10.1038/s41467-022-33221-z)
Supplement: Supplementary file 3 — Reporting Summary [file 41467_2022_33221_MOESM3_ESM.pdf]

nature portfolio

Corresponding author(s): Dr. Lucy shapiro, Dr. Aaron D. Gitler, Dr. Keren Lasker

Last updated by author(s): Sep 6, 2022

Reporting Summary

Nature Portfolio wishes to improve the reproducibility of the work that we publish. This form provides structure for consistency and transparency in reporting. For further information on Nature Portfolio policies, see our Editorial Policies and the Editorial Policy Checklist.

Statistics

For all statistical analyses, confirm that the following items are present in the figure legend, table legend, main text, or Methods section.

n/a	Confirmed	
☐	☒	The exact sample size (*n*) for each experimental group/condition, given as a discrete number and unit of measurement
☐	☒	A statement on whether measurements were taken from distinct samples or whether the same sample was measured repeatedly
☐	☒	The statistical test(s) used AND whether they are one- or two-sided<br>*Only common tests should be described solely by name; describe more complex techniques in the Methods section.*
☒	☐	A description of all covariates tested
☒	☐	A description of any assumptions or corrections, such as tests of normality and adjustment for multiple comparisons
☐	☒	A full description of the statistical parameters including central tendency (e.g. means) or other basic estimates (e.g. regression coefficient) AND variation (e.g. standard deviation) or associated estimates of uncertainty (e.g. confidence intervals)
☐	☒	For null hypothesis testing, the test statistic (e.g. *F*, *t*, *r*) with confidence intervals, effect sizes, degrees of freedom and *P* value noted<br>*Give P values as exact values whenever suitable.*
☒	☐	For Bayesian analysis, information on the choice of priors and Markov chain Monte Carlo settings
☒	☐	For hierarchical and complex designs, identification of the appropriate level for tests and full reporting of outcomes
☐	☒	Estimates of effect sizes (e.g. Cohen's *d*, Pearson's *r*), indicating how they were calculated

Our web collection on statistics for biologists contains articles on many of the points above.

Software and code

Policy information about availability of computer code

Data collection	Phase contrast and fluorescence microscopy imaging of Caulobacter cells: Inverted fluorescence microscope (Leica DMi8) equipped with Hamamatsu EMCCD camera and oil immersion objective with a 100x/1.4NA.<br>Confocal imaging and photobleaching: Inverted LSM 710 laser-scanning confocal microscope (Zeiss) with an oil immersion objective with a 63x/l.4NA.<br>Correlative Cryo-Fluorescence imaging: CorrSight inverted microscope (ThermoFisher Scientific) using EC Plan-Neofluar Sx/0.16NA and EC Plan-Neofluar 40x/0.9NA air objectives (Carl Zeiss Microscopy) and a 1344x102 4 px ORCA-Flash 4.0 camera (Hamamatsu)<br>Cryo electron tomography:Titan Krios (ThermoFisher Scientific) operating at 300 kV accelerating voltage with a Gatan K2 Summit camera equipped with a Quantum energy filter. Tilt series were obtained using SerialEM-v3.8b11.
Data analysis	Bioinformatics analysis: Geneious Prime-v2020.0.4, NetSurfP-v2.0, Jpred-v4.0.0, and python 3.7.3<br>Caulobacter cells image analysis: ImageJ/FIJI version 1.53c with the MicrobeJ plugin version 5.131(22) followed by custom scripts in Matlab 202 0a, found at https://zenodo.org/record/7042738#.YxG3P-zMI6A. IDR and full protein simulations:ABSINTH abs3.2_opls.prm implicit solvent model and CAMPARI V2 Monte Carlo simulation, SOURSOP 0.1.9, and MDTraj 1.9.5 . CryoET reconstruction, analysis, and segmentation: MotionCor2, IMOD-v4.10.28, Dynamo-v1.1.514, AMIRA v6.7, and TomoSegMemTV vApr2020<br>Curve fitting and statistical analysis: Prism 9.3.1, Excel, part of Microsoft 365 version 2007. All other scripts used in this study are available from the corresponding authors upon request.

For manuscripts utilizing custom algorithms or software that are central to the research but not yet described in published literature, software must be made available to editors and reviewers. We strongly encourage code deposition in a community repository (e.g. GitHub). See the Nature Portfolio guidelines for submitting code & software for further information.

Data

Policy information about availability of data

All manuscripts must include a data availability statement. This statement should provide the following information, where applicable:

- Accession codes, unique identifiers, or web links for publicly available datasets
- A description of any restrictions on data availability
- For clinical datasets or third party data, please ensure that the statement adheres to our policy

Proceeded tomograms are deposited to the Electron Microscopy Data Resource with accession codes EMD-23622, EMD-23623, and EMD-23624. Raw cryo-EM data have been deposited with the Electron Microscopy Public Image Archive with accession codes: EMPIAR-10693, EMPIAR-10688, and EMPIAR-10689. Bioinformatics analysis, phase diagram data, microscopy images, and all-atom simulation data for Monte Carlo simulations are provided at https://zenodo.org/record/7042738#.YxG3P-zMI6A90. Source data are provided with this work.
All other data supporting the findings of this study are included in the main text and the supplementary information. Strains are available from the corresponding author(s) upon request.

Human research participants

Policy information about studies involving human research participants and Sex and Gender in Research.

Reporting on sex and gender	*Use the terms sex (biological attribute) and gender (shaped by social and cultural circumstances) carefully in order to avoid confusing both terms. Indicate if findings apply to only one sex or gender; describe whether sex and gender were considered in study design whether sex and/or gender was determined based on self-reporting or assigned and methods used. Provide in the source data disaggregated sex and gender data where this information has been collected, and consent has been obtained for sharing of individual-level data; provide overall numbers in this Reporting Summary. Please state if this information has not been collected. Report sex- and gender-based analyses where performed, justify reasons for lack of sex- and gender-based analysis.*
Population characteristics	*Describe the covariate-relevant population characteristics of the human research participants (e.g. age, genotypic information, past and current diagnosis and treatment categories). If you filled out the behavioural & social sciences study design questions and have nothing to add here, write "See above."*
Recruitment	*Describe how participants were recruited. Outline any potential self-selection bias or other biases that may be present and how these are likely to impact results.*
Ethics oversight	*Identify the organization(s) that approved the study protocol.*

Note that full information on the approval of the study protocol must also be provided in the manuscript.

Field-specific reporting

Please select the one below that is the best fit for your research. If you are not sure, read the appropriate sections before making your selection.

☒ Life sciences ☐ Behavioural & social sciences ☐ Ecological, evolutionary & environmental sciences

For a reference copy of the document with all sections, see nature.com/documents/nr-reporting-summary-flat.pdf

Life sciences study design

All studies must disclose on these points even when the disclosure is negative.

Sample size	No predetermination of sample size was done. Sample sizes were chosen such that significant statistical information (mean, standard error of mean, and P values) could be derived. The exact sample sizes (e.g., number of cells, number of condensates) are noted in figure legends.
Data exclusions	No data were excluded.
Replication	All experiments were repeated (biological replicates) at least twice. All attempts of replication were successful.
Randomization	In our FRAP and partitioning studies condensates were randomly selected without biases. For cell shape and size statistics, bacterial cells were randomly selected without biases. For bioinformatics and simulation studies, data analysis was performed on the entire dataset
Blinding	Blinding is not relevant to the study. Researchers were not blinded to the identity of the samples as it was not technically or practically feasible to do. The same pipeline was used across strains for each assay, compensating for the lack of blinding.

Reporting for specific materials, systems and methods

We require information from authors about some types of materials, experimental systems and methods used in many studies. Here, indicate whether each material, system or method listed is relevant to your study. If you are not sure if a list item applies to your research, read the appropriate section before selecting a response.

Materials & experimental systems

n/a	Involved in the study
☒	☐ Antibodies
☐	☒ Eukaryotic cell lines
☒	☐ Palaeontology and archaeology
☒	☐ Animals and other organisms
☒	☐ Clinical data
☒	☐ Dual use research of concern

Methods

n/a	Involved in the study
☒	☐ ChIP-seq
☒	☐ Flow cytometry
☒	☐ MRI-based neuroimaging

Eukaryotic cell lines

Policy information about cell lines and Sex and Gender in Research

Cell line source(s)	U20S cells from ATCC
Authentication	Cell lines were not authenticated
Mycoplasma contamination	Cell lines were not tested for mycoplasma contamination
Commonly misidentified lines (See ICLAC register)	No commonly misidentified cell lines were used in the study
