# Supplementary figures and images for: The material properties of a bacterial-derived biomolecular condensate tune biological function in natural and synthetic systems
Source: Nat Commun. 2022 Sep 26;13:5643. doi: 10.1038/s41467-022-33221-z (PMC9512792; doi:10.1038/s41467-022-33221-z)

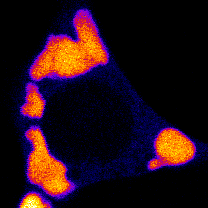

Supplement: Supplementary file 6 — Supplementary Video 1 [file 41467_2022_33221_MOESM6_ESM.gif]
